# RNA Stimulates Aurora B Kinase Activity during Mitosis

**DOI:** 10.1371/journal.pone.0100748

**Published:** 2014-06-26

**Authors:** Ashwini Jambhekar, Amy B. Emerman, Caterina T. H. Schweidenback, Michael D. Blower

**Affiliations:** 1 Department of Molecular Biology, Massachusetts General Hospital, Boston, Massachusetts, United States of America; 2 Department of Genetics, Harvard Medical School, Boston, Massachusetts, United States of America; Duke University Medical Center, United States of America

## Abstract

Accurate chromosome segregation is essential for cell viability. The mitotic spindle is crucial for chromosome segregation, but much remains unknown about factors that regulate spindle assembly. Recent work implicates RNA in promoting proper spindle assembly independently of mRNA translation; however, the mechanism by which RNA performs this function is currently unknown. Here, we show that RNA regulates both the localization and catalytic activity of the mitotic kinase, Aurora-B (AurB), which is present in a ribonucleoprotein (RNP) complex with many mRNAs. Interestingly, AurB kinase activity is reduced in *Xenopus* egg extracts treated with RNase, and its activity is stimulated *in vitro* by RNA binding. Spindle assembly defects following RNase-treatment are partially rescued by inhibiting MCAK, a microtubule depolymerase that is inactivated by AurB-dependent phosphorylation. These findings implicate AurB as an important RNA-dependent spindle assembly factor, and demonstrate a translation-independent role for RNA in stimulating AurB.

## Introduction

The spindle is a macromolecular assembly of microtubules and associated proteins that controls the segregation of chromosomes during each eukaryotic cell division. During mitosis, centrosomes nucleate microtubules that search for and capture chromosomes, resulting in chromosome alignment at the metaphase plate [1]. In addition, chromatin stimulates microtubule nucleation, resulting in spindle self-organization in the absence of centrosomes [2], [3]. The importance of each of these pathways varies between cell types [4]. Most somatic cells use centrosomes to promote spindle assembly, while female meiotic cells lack centrosomes and instead use chromatin-dependent pathways to nucleate and assemble spindles.

The two major chromatin-dependent pathways regulating microtubule assembly involve the small GTPase Ran and the mitotic kinase Aurora-B (AurB). Ran-GTP is produced near chromosomes and releases spindle assembly factors–such as NuMA [5], [6], TPX2 [7], HURP [8], [9], and NuSAP [10] –from inhibition by the nuclear import receptor Importin β. In parallel, the Chromosome Passenger Complex (CPC)–consisting of the kinase AurB and associated proteins Incenp, Borealin (DasraA in *Xenopus*), and Survivin–localizes to chromatin, centromeres, and microtubules during mitosis to promote spindle assembly (reviewed in [11]–[13]). The major targets of this complex are two microtubule-depolymerizing proteins: Op18 [14], which binds to and sequesters free tubulin dimers [15], and MCAK [16], [17], a kinesin-13 family member [18] that is the major depolymerizing activity in *Xenopus* egg extracts [19], [20]. Both proteins are inactivated when phosphorylated by AurB during mitosis [14], [16], [17], [21], allowing robust spindle assembly.

Although many proteins regulating spindle assembly have already been characterized, recent work has demonstrated that RNA also plays a critical role in promoting proper spindle assembly. Many mRNAs encoding mitotic regulators and components of the translation machinery localize to mitotic and meiotic spindles [22]–[30], and studies in several systems have demonstrated that RNA and RNA-binding proteins are important for proper mitotic progression [31]–[34]. Previous work demonstrated a role for spindle-localized translation in regulating cell division [23], [24], and our group found that RNA was required in a translation-independent manner for spindle assembly in meiotic *Xenopus* egg extracts [22], [35].

Our previous study indicated that a Rae1-containing RNP regulated spindle assembly in *Xenopus* and human cells [35]. However, whether RNA regulates additional mitotic spindle assembly factors, and the mechanism by which RNA promotes spindle assembly in a translation-independent manner, remain unknown. Interestingly, recent work in cultured mouse and human cells demonstrated that AurB binds to and is activated by a transcript arising from minor satellite DNA [36], and that the localization of both Incenp and Survivin to the centromere is abolished in RNase-treated chromosome spreads [37], suggesting that the CPC could be regulated by binding to RNA. However, the mechanism of CPC regulation by RNA and the functional consequences of this interaction are unclear. Here we use *Xenopus* egg extracts to explore the hypothesis that the CPC is another spindle assembly factor that is regulated by RNA binding. We show that AurB activity is reduced in RNase-treated extracts, and that AurB is present in a complex with RNAs. We also show that the CPC binds to RNA *in vitro,* leading to AurB activation. Thus, we propose that AurB is an additional spindle regulatory enzyme governed by RNA-binding.

## Results

### RNA activates the mitotic kinase AurB

**Figure 1:**
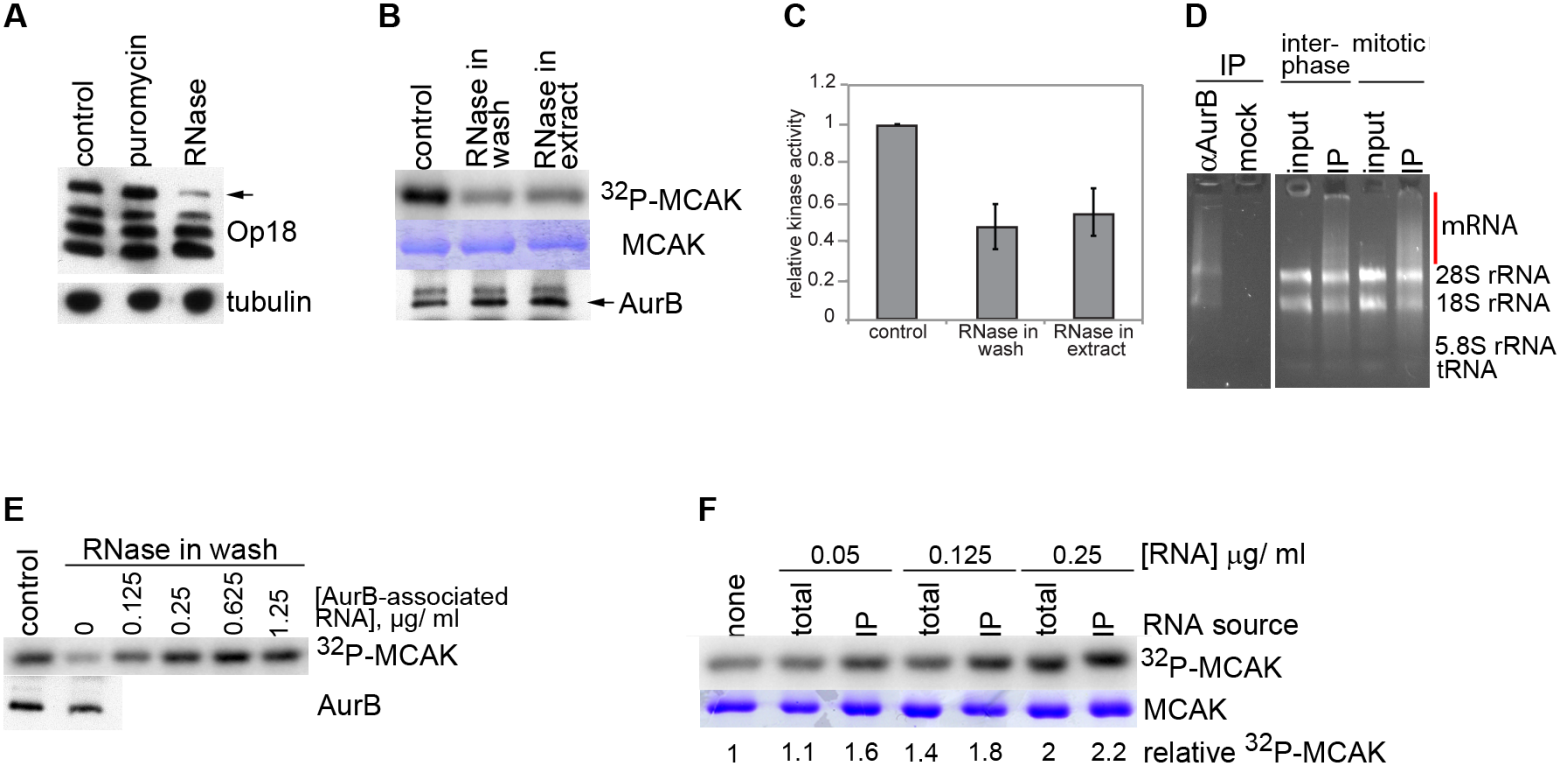
AurB is an RNA-dependent kinase. **A)** Hyperphosphorylation of Op18 in control extracts, or extracts treated with puromycin or RNase, containing sperm nuclei at a concentration of 5000 nuclei/µl. Arrow indicates AurB-dependent hyperphosphorylated form of Op18, as detected by western blot. Tubulin is shown as a loading control. **B)** Phosphorylation of MCAK N-terminus by AurB immunoprecipitated from control or RNase-treated extracts containing sperm nuclei, and washed +/− RNase following IP. MCAK substrate and AurB amounts are shown as loading controls. **C)** Quantitation of signals shown in (B). n = 4–5 experiments, error bars are SEM. **D)** RNA isolated from mock or αAurB IPs. RNA from input (total) extract is shown as a control. RNA was isolated from interphase or mitotic extracts. Red bar highlights transcripts enriched in AurB IPs. **E)** Rescue of RNase-washed AurB kinase activity by transcripts associated with AurB following stimulation with chromatin. Immunoprecipitations were split into aliquots containing equal amounts of beads during the final wash. One aliquot of each sample was analyzed by western blot as a loading control (lower panel), and remaining aliquots were used for kinase reactions (upper panel). **F)** Rescue of kinase activity as in (C) with total or AurB-binding RNAs. E-F) Gels are representative of experiments performed at least in duplicate.

Previous work in mouse and human cells suggested that localization and activation of AurB is regulated by RNA. To determine if AurB activity is regulated by RNA in *Xenopus* egg extracts, we assayed AurB activity by monitoring Op18 hyperphosphorylation [14]. We observed consistently reduced hyperphosphorylation in RNase-treated extracts, both in the presence or absence of phosphatase inhibitors, but not in extracts treated with protein synthesis inhibitors (Fig. 1A, S1A), consistent with a translation-independent role for RNA in activating AurB. To measure the kinase activity of AurB directly, we immunoprecipitated AurB using custom antibodies (Fig. S1B) and tested its ability to phosphorylate an N-terminal fragment of the AurB target MCAK [16], [17] *in vitro* in the presence or absence of RNA (Fig. 1B–C, Fig. S1C). RNase treatment was conducted either in extract prior to immunoprecipitation, or in the wash following AurB isolation on beads. These treatments reduced AurB activity to less than half of control levels (Fig. 1C). Similar results were obtained using histone H3, another target of AurB [38], as the substrate with either endogenous or recombinant AurB in extracts (Fig. S1D–E). The reduction in activity following RNase treatment in the wash is notable, as it suggests that RNA is present in a complex with AurB, and not simply required for steps upstream of kinase activation. Importantly, we found that RNaseA did not copurify with AurB under our IP conditions, demonstrating that the inhibitory affect of RNase treatment is not an indirect affect of RNase binding to AurB (Fig. S1I). To test whether the reduction of kinase activity is due to translation inhibition, AurB activity was assessed in extracts treated with puromycin at concentrations that inhibit both cap- and IRES-dependent translation (Fig. S1F). AurB isolated from puromycin-treated extracts displayed activity similar to that from untreated extracts, indicating that RNA stimulates AurB activity independently of translation (Fig. S1G). As expected, AurB from interphase extracts displayed low activity [39]. We have previously shown that RNase treatment reduces CyclinB-CDK kinase activity by ∼25%, and that this decrease results from inhibiting translation [35]. Therefore, the translation-independent regulation of catalytic activity by RNA is not a hallmark of all kinases, and suggests that AurB is particularly sensitive to the presence of RNA.

To determine if AurB associated with RNA in egg extracts, we isolated AurB under different states of kinase activation–inactive (interphase) and active (mitotic) (Fig. S1G)– and assayed for the presence of RNA coprecipitating with AurB. AurB coprecipitated roughly equivalent amounts of RNA under both conditions, indicating that binding to RNA, like the assembly of the CPC components [39], is not cell-cycle regulated (Fig. 1D). Under immunoprecipitation conditions leading to >90% AurB depletion from extract, 0.4% of RNA from extract coprecipitated with AurB, whereas only 0.01% copurified with nonspecific IgG antibodies. We tested whether transcripts associated with AurB could rescue the decrease in kinase activity due to RNase treatment. AurB was stimulated in extracts by addition of chromatin, AurB-bound RNA was purified (Fig. S1H), and the RNA was added to *in vitro* IP-kinase reactions that had been treated with RNase in the washes and further washed to remove RNase (Figure S1I–J). The pool of AurB-associated, purified RNA could rescue kinase activity in a concentration-dependent manner (Fig. 1E), but had no effect on control, untreated reactions (Fig. S1K). Total RNA from extract could also rescue kinase activity (Fig. 1F), albeit at higher concentrations, indicating that the rescue was more efficient with AurB-bound transcripts. The ability to rescue kinase activity by adding RNA demonstrates that the loss of activity following RNase treatment does not arise from non-specific toxicity of the treatment. These results indicate that RNA is a component of an AurB-containing complex and promotes kinase activation.

### The CPC binds RNA in a sequence-specific manner

**Figure 2:**
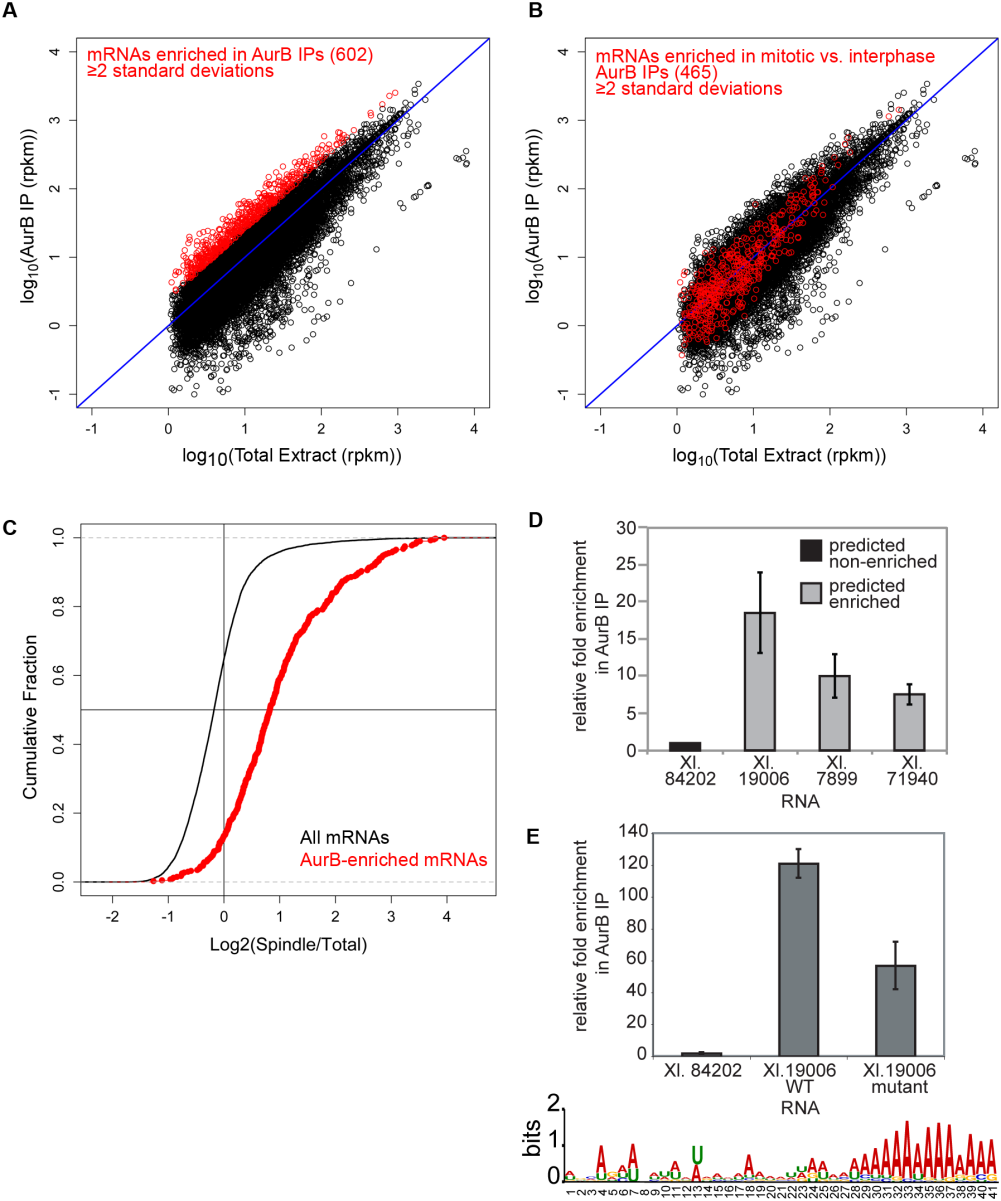
Active AurB binds a distinct subset of transcripts that overlaps with RNAs found on spindles, and a degenerate primary sequence RNA motif mediates AurB recognition. A) Scatter plot showing enrichment of a subset of transcripts (red) in mitotic AurB immunoprecipitations from biological triplicates. B) Scatter plot showing transcripts bound to AurB in interphase extracts. Transcripts specifically bound in metaphase but not in interphase are indicated with red points. C) Cumulative distribution curve showing fraction of total or AurB-bound transcripts as a function of their enrichment on spindles. D) qPCR detection of transcripts predicted by sequencing analysis to be enriched or not enriched in AurB immunoprecipitations. (n = 3 extracts). E) Bottom: Sequence logo [77] showing a poly-A motif enriched in AurB-bound RNAs, but not in transcripts from total RNA. Top: Binding of AurB to *in vitro* transcribed fragments of Xl. 19006 containing the WT motif (“WT”) or harboring mutations in the 41 nt motif (“mutant”), with each purine mutated to the other purine, and each pyrimidine mutated to the other pyrimidine (see Methods). Binding to *in vitro* transcribed Xl. 84202 is shown as a negative control. Transcripts were added to extract, and AurB was immunoprecipitated as in (C) n = 3 extracts. (D–E) All error bars represent SEM.

AurB-associated RNA qualitatively differed from total extract RNA, with an apparent decrease in the proportion of rRNA and an increased proportion of high molecular weight RNAs present in AurB immunoprecipitations (Fig. 1D, line). To determine if AurB associates with specific transcripts, we isolated and sequenced transcripts bound to AurB in interphase and mitosis. Over 600 RNAs were enriched in mitotic AurB immunoprecipitations compared to their levels in total extract (Fig. 2A, red points); 465 were specifically enriched in mitotic–but not interphase–immunoprecipitations, suggesting cell-cycle regulated binding of specific RNA targets (Fig. 2B, red points). Many of these transcripts contained clear protein-coding domains, although we cannot exclude the possibility that some of the transcripts may be non-coding due to incomplete sequencing and annotation of the *X. laevis* genome. Gene Ontology analysis [40] of AurB-enriched transcripts demonstrated that they were significantly enriched for mRNAs coding for cytoskeletal proteins and transcription factors (Table S1), similar to mRNAs that we have previously reported to be enriched on microtubules (Table S1). In contrast to the association of AurB with satellite repeats in mouse cells [36], we did not observe a preferential association of AurB with repetitive transcripts (Cenp-A-associated repeats [41], transposons, or other repeats; data not shown). Interestingly, transcripts enriched in mitotic AurB immunoprecipitations showed a striking correlation with transcripts localized to spindles (Fig. 2C and Table S3)(Pearson correlation coefficient = 0.42, p-value for spindle enrichment of AurB-enriched transcripts vs all transcripts = 2.2×10^−16^), demonstrating that AurB associates with mRNAs that are enriched on mitotic spindles. The binding of three transcripts enriched in mitotic AurB immunoprecipitations was confirmed by qPCR (Fig. 2D) in three extracts. Transcripts bound to AurB in mitosis showed significant over-representation of adenines (Table S2), and motif analysis of the primary sequences using MEME [42] revealed that transcripts bound to AurB in mitosis were enriched for A-rich tracts (poly-A tails were excluded from analysis). Mutation of each purine to the other purine, and each pyrimidine to the other pyrimidine, within this region in one AurB-associated RNA reduced binding by half (Fig. 2E). This region may either be recognized directly by the AurB complex, or it may influence formation of an RNA structure at a distal site that is recognized by the complex. The number and sequence specificity of transcripts bound to AurB are reminiscent of the transcripts bound by many other RNA-binding proteins that have been examined by high-throughput sequencing [43]–[47], suggesting that AurB is a *bona fide* RNP component.

### RNA directly binds and activates the CPC

**Figure 3:**
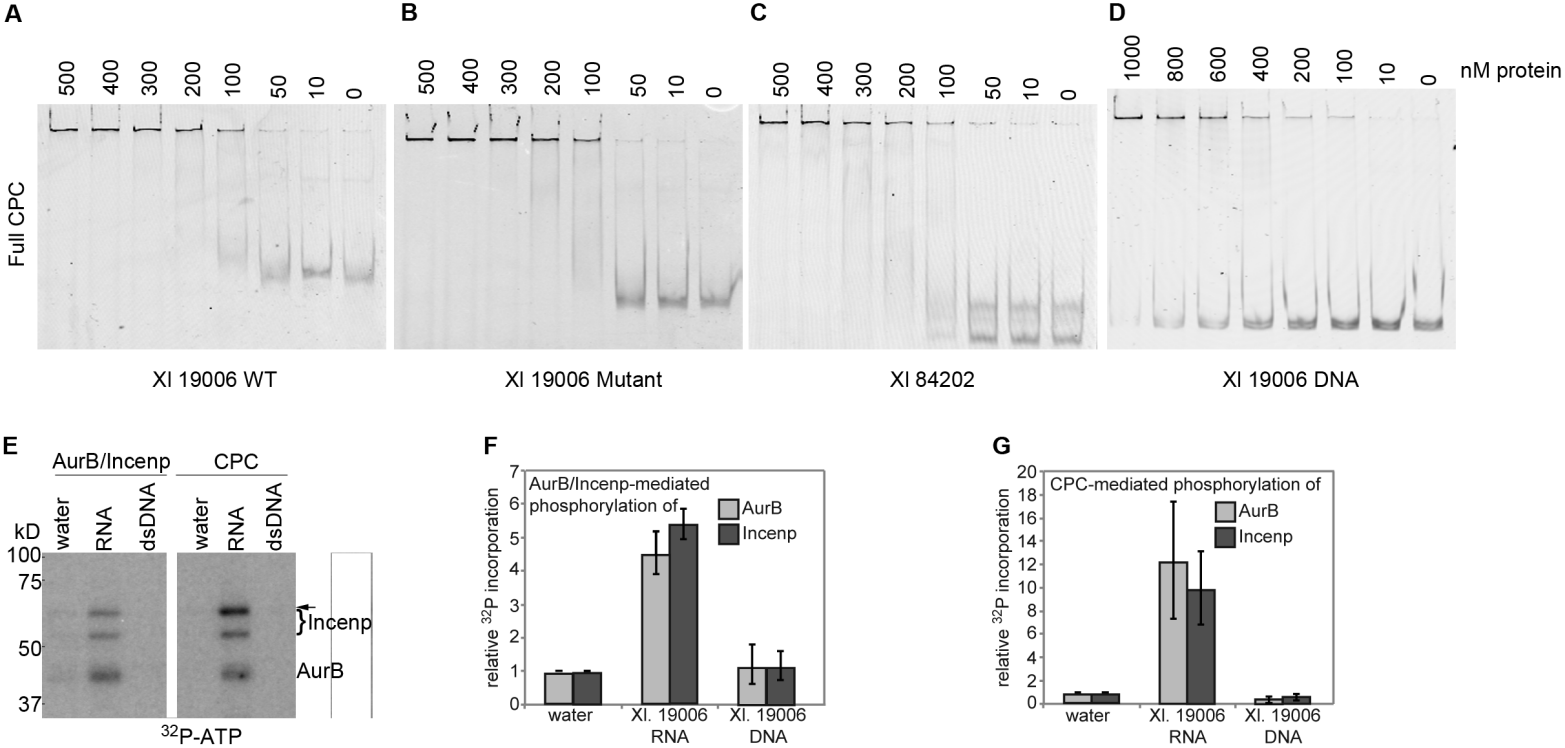
The CPC binds to and is stimulated by RNA *in vitro*. Binding of full, recombinant CPC to **A)** AurB-interacting Xl. 19006 RNA, **B)** Xl. 19006 RNA lacking the poly-A motif shown in Fig. 2, **C)** negative control Xl. 84202 RNA, **D)** PCR product encoding the RNA shown in (A). **E)** Phosphorylation of Incenp and AurB *in vitro* by purified AurB/Incenp or full CPC in the presence or absence of RNA or double-stranded DNA. Incenp band marked with arrow is quantified in F. Single gel image is shown, with intervening lanes omitted. **F–G)** Quantification of signals indicated in (E) from 3 independent experiments. Error bars represent SEM.

Our experiments in extracts did not define whether RNA binds directly to the CPC, or binds indirectly via other proteins that may associate with the complex. To determine whether the CPC binds directly to RNA, we examined the interaction of recombinant CPC with RNA *in vitro* (Fig. S2A, B) using electrophoretic mobility shift assays (EMSA). We tested binding to three transcripts–Xl. 19006, Xl. 19006 mut, and Xl. 84202 (Fig. 2D)–that display varying affinities for the CPC in extract (Fig. 2D and Table S3). The CPC bound equivalently to all three RNAs *in vitro*, but bound with a much lower affinity to a DNA fragment encoding Xl. 19006 (Fig. 3A–D, Fig. S3E, Table 1). The interaction of the CPC with RNA *in vitro* is characterized by a lack of sequence specificity and a lack of charge-mediated interactions (as evidenced by a lower affinity of DNA), which is reminiscent of the mode of interaction of PRC2 with RNA *in vitro* [48]. This result suggests that the CPC may interact with RNA through relatively nonspecific base stacking interactions, as exemplified by the recent structure of the exosome [49]. These results indicate that the CPC possesses RNA-binding activity, in addition to the chromatin- and histone-binding properties described previously [50] [51].

**Table 1:** Apparent dissociation constants (nM) for binding of various CPC subunits to RNA and DNA sequences as defined in Fig. 3.

	19006 WT	19006 Mutant	84202	DNA
**GFP-AurB**	160+/−89	118+/−82	175+/−55	557+/−400
**Survivin**	1114+/−451	1167+/−379	1569+/−469	Not Measurable
**Survivin/DasraA**	73+/−22	67+/−49	74+/−87	5449+/−4017
**AurB/Incenp**	70+/−39	59+/−27	112+/−59	1122+/−1218
**CPC**	53+/−8	38+/−23	56+/−44	1348+/−1437

To further define the RNA-binding subunits within the CPC, we tested RNA binding of AurB alone or in complex with Incenp, and Survivin alone or in complex with DasraA. Full-length AurB alone displayed appreciable affinity for RNA (Table 1, Fig. S2C, S3A), whereas the N-terminus of AurB did not (data not shown). Survivin bound to RNA when complexed with DasraA, but not on its own (Fig. S2D, S3B). Interestingly, the AurB/Incenp complex demonstrated some sequence-specific binding, with a 2-fold higher affinity for Xl. 19006 (which binds strongly to AurB in extract (Fig. 2D)), compared to that for Xl. 84202 (which binds minimally in extract (Fig. 2D)) (Fig. S2F, S3D). No protein combinations showed specificity for the wild-type Xl. 19006 sequence over the mutant version (Fig. 2D), indicating that sequence-specific binding results from additional regulation *in vivo.* Taken together, our results demonstrate that the CPC is a *bona fide* RNA binding-complex with at least 2 RNA-binding domains: one in AurB and another in Survivin/DasraA (likely in DasraA).

To determine if RNA-binding by the CPC activates AurB we adapted a protocol [52] in which the activity of recombinant AurB is first abolished by λ-phosphatase treatment, followed by inactivation of the phosphatase. Reactivation of AurB in the presence of stimulatory factors was assessed by measuring Incenp phosphorylation and AurB autophosphorylation. In this assay, RNA stimulated the activity of both AurB/Incenp and the full CPC (Fig. 3E–G), whereas double-stranded DNA of the same sequence did not. RNA did not stimulate the catalytic activity of GFP-Aurora-B lacking Incenp, demonstrating that RNA must act in combination with other factors to promote AurB activation (data not shown). These results demonstrate that RNA binds directly to the CPC and stimulates the catalytic activity of AurB, and that the effect does not result from simple electrostatic charge-based interactions. As expected based on the EMSA studies above, two different RNA sequences (Xl. 84204 and Xl. 19006) equally stimulated kinase activity of the purified CPC *in vitro* (Fig. S4A). Two different mechanisms for enhancement of catalytic activity by RNA seem plausible: RNA may bind and allosterically activate the enzyme, or it may serve as a scaffold to cluster multiple kinase complexes, as has been reported for chromatin and microtubules [51], [53]. We favor the former possibility for two reasons: first, *in vitro* kinase assays were performed under saturating RNA conditions in which the majority of RNA molecules are expected to bind a single CPC. Second, *in vitro* reactions containing an 80% reduction in the amount of RNA, which is predicted to enhance clustering by increasing the number of CPC molecules bound per transcript, caused no change in *in vitro* autophosphorylation (data not shown).

### RNA promotes proper AurB and MCAK localization at centromeres

**Figure 4:**
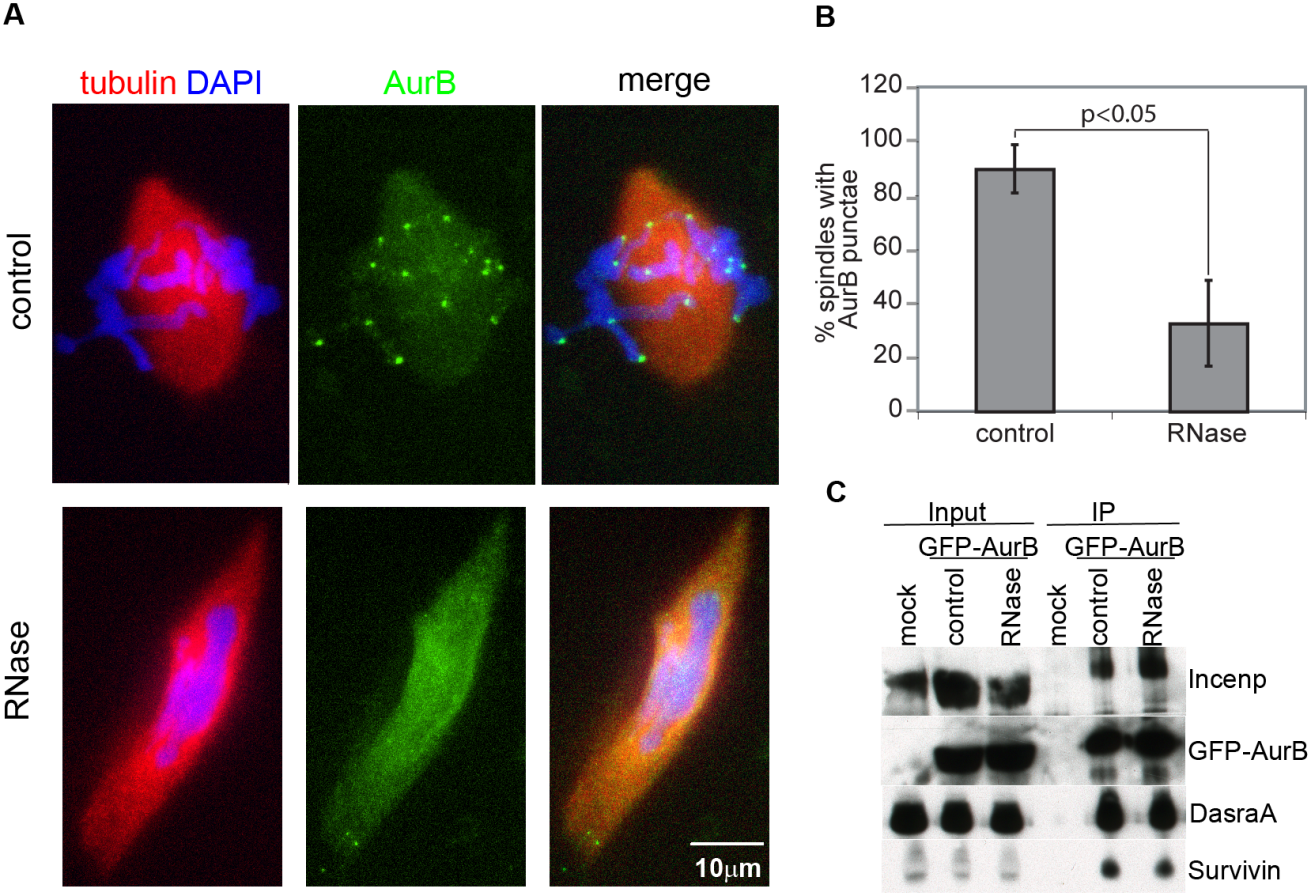
RNA is required for AurB localization to centromeres. **A)** Localization of GFP-AurB to centromeres in control and RNase-treated spindles. Images shown are projections. **B)** Percentage of spindles showing chromosomal AurB punctae. Spindles were scored as positive if at least 6 punctae were visible. (n = 3 extracts, 50–100 spindles per extract, p<0.05 by paired t-test). Error bars represent SEM. **C)** Coprecipitation of the core CPC members–Incenp, DasraA, and Survivin–with GFP-AurB from control or RNase-treated extracts. Immunoprecipitation from extract lacking GFP-AurB (“mock”) is shown as a control. Input amounts are shown. Results are representative of experiments performed at least in triplicate.

Because AurB localization and activation are controlled by overlapping mechanisms (reviewed in [11]) we analyzed AurB localization at inner centromeres by adding recombinant, GFP-tagged AurB to spindle assembly reactions. While control spindles displayed multiple, bright AurB foci at centromeres, the number and intensity of such foci were greatly reduced upon RNase-treatment (Fig. 4A–B). AurB remained localized diffusely to chromatin and microtubules in RNase-treated extracts, indicating that centromere enrichment was differentially compromised. The localization defect did not result from disruption of CPC assembly, as the core CPC subunits associated similarly with GFP-AurB in control and RNase-treated extracts (Fig. 4C). These results reveal that RNA functions analogously to the protein components of the CPC, which govern both AurB enrichment at centromeres and kinase activity *in vitro* (reviewed in [11]).

**Figure 5:**
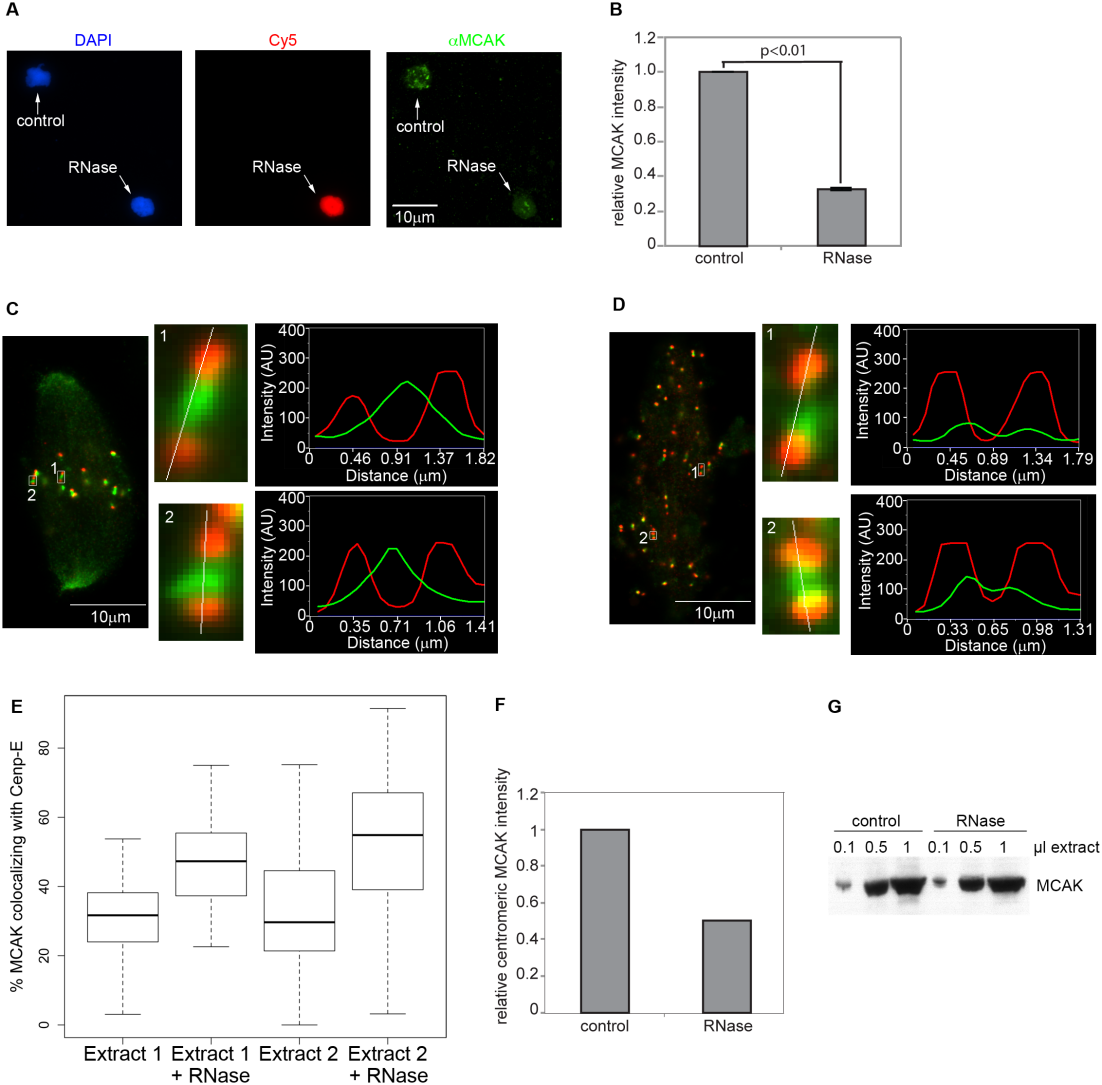
MCAK localization to centromeres is defective in RNase-treated extracts. **A)** MCAK localization was assessed on nuclei cycled through interphase and rearrested in metaphase in the presence of nocodazole. Control or RNase-treated nuclei (the latter labeled with Cy5-dUTP) were fixed, copelleted onto a single coverslip, and processed for αMCAK immunofluorescence. Pairs of control and RNase-treated nuclei in the same field of view were assayed for MCAK localization. Images shown are projections, except for Cy5, which is a single plane. **B)** Quantitation of centromeric MCAK levels from (A). (n = 3 extracts, 14–20 pairs of nuclei per extract, p<0.01 by single-sample t-test of average normalized values from each extract). Error bars represent SEM. **C)** Localization of Cenp-E (red) and MCAK (green) to sister kinetochores in control spindles. Insets show single pairs of kinetochores magnified 12x, and a line scan of Cenp-E and MCAK intensities along the indicated trajectories. **D)** Same as in (C), but RNase-treated. MCAK signal has been enhanced in kinetochore insets. C–D) Images shown are single planes. Kinetochore insets are magnified 12X. **E)** Quantitation of data shown in (C–D). Box plot showing the distribution of MCAK overlap with Cenp-E in two independent extracts. Box represents the median and upper and lower quartiles. Bars show the extent of outliers in each condition. 21–31 kinetochore pairs were measured in each extract. **F)** Quantitation of centromeric MCAK levels from (C–D). (n = 2 extracts, 5–6 spindles of each category per extract). **G)** Western blot showing MCAK amounts in control or RNase-treated extracts.

Our results established that RNA is required for maximal levels of AurB activity and localization. Because AurB phosphorylation is required for centromere- and chromatin- localization of MCAK [17], [54], we asked whether MCAK localization was affected in RNase-treated extracts. We first assessed localization on nuclei that had been cycled through interphase and rearrested in mitosis in the presence of nocodazole to depolymerize microtubules, which has previously been used to assess the role of AurB in MCAK localization [54]. This treatment ensured that any effects of RNase treatment were not due to differences in kinetochore-microtubule attachments, which also affect MCAK intensity and distribution at centromeres [55]. Under these conditions, MCAK localization to centromeres was consistently reduced in the absence of RNA (Fig. 5A–B). The reduction in MCAK localization was not the result of a decrease in total MCAK levels following RNase treatment (Figure 5G). Next, we tested MCAK localization relative to that of the outer kinetochore marker Cenp-E in the context of cycled spindles. Consistent with our results in nocodazole-treated spindles we observed that MCAK intensity at centromeres was reduced (Figure 5F). Additionally, the remaining MCAK staining at centromeres was redistributed compared to that of untreated spindles. While MCAK formed a single focus between each pair of sister kinetochore Cenp-E foci on control spindles, upon RNase treatment MCAK redistributed away from the centromere and formed two foci closer to the Cenp-E markers (Fig. 5C–D). This relocalization was apparent as an increased overlap between Cenp-E and MCAK foci (Fig. 5E), and occurred irrespectively of inter-kinetochore distances (Fig. 5C–D, insets), suggesting that the phenomenon does not arise secondarily to defects in tension between kinetochores. These results are consistent with the phenotypes reported for two non-phosphorylatable mutants of MCAK lacking AurB phosphorylation sites. In *Xenopus* egg extracts, non-phosphorylatable MCAK displayed reduced centromeric localization [21], while in human cells a similar mutant redistributed closer to the outer kinetochore [16]. These results demonstrate that RNA regulates MCAK localization, likely via its effects on AurB.

### MCAK inhibition rescues spindle assembly in the absence of RNA

**Figure 6:**
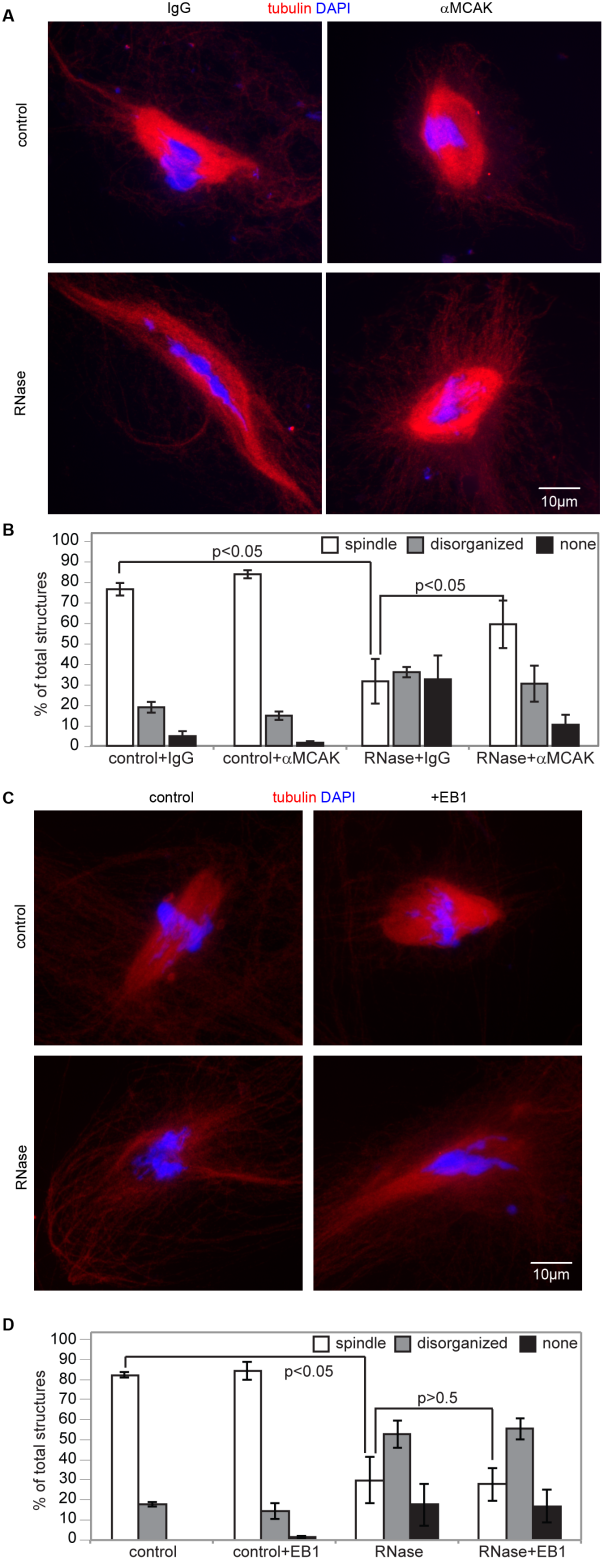
Spindle defects in the absence of RNA arise from aberrant regulation of MCAK. **A)** Spindles formed in extracts (+/− RNase) in the presence of nonspecific or αMCAK antibodies. An example of a rare spindle in control RNase-treated extracts is shown. All spindle reactions contained CyclinB Δ90. Images shown are projections. **B)** Spindle assembly efficiency in extracts shown in (A). “None” indicates that no microtubule structures formed around nuclei. (n = 4 extracts, >100 nuclei per extract, p<0.05 by paired t-test). Error bars represent SEM. **C)** Examples of spindle assembly in control and RNase-treated extracts in the presence or absence of 750 nM EB1-GFP. Disorganized structures formed in RNase-treated extracts are shown. Images shown are projections. **D)** Spindle assembly efficiency in extracts shown in (C). (n = 3 extracts, >100 nuclei per extract p<0.05 for control vs RNase-treated p>0.5 for RNase +/− EB1 by paired t-test). Error bars represent SEM.

If RNA does, in fact, activate AurB *in *vivo, one expectation is that the microtubule depolymerization activity of MCAK, which is repressed by AurB-mediated phosphorylation during mitosis [17], [21], [54], would be hyperactive in the absence of RNA. Although AurB has many targets during mitosis, including kinetochore proteins whose phosphorylation regulates microtubule attachment [56], MCAK is the primary target of AurB in *Xenopus egg* extracts [17], [57]. Therefore, inhibiting MCAK activity would be predicted to ameliorate the spindle defects observed upon RNase treatment of extracts. To test this hypothesis, we assessed the effects of antibody inhibition of MCAK on spindle assembly. RNase-treatment of extracts resulted in reduced efficiency of spindle assembly, with the spindles that did form exhibiting elongated pole-to-pole distances (Fig. 6A–B, Fig. S1L). These effects occurred independently of translation [22], [35]. Our results suggested that the spindle phenotypes in RNase-treated extracts resulted from partial AurB inhibition, as well as from effects on other spindle assembly factors such as Ran and Rae1. Strikingly, addition of low levels of inhibitory αMCAK antibodies (7% of the amount needed for complete inhibition of MCAK [58]) improved the efficiency of spindle assembly (Fig. 6B), as well as the overall morphology (Fig. S4B). This result indicates that RNase treatment does not produce a globally toxic environment incapable of supporting microtubule polymerization. It is formally possible that RNA acts to inhibit MCAK independently of AurB resulting in hyperactivation of MCAK in RNase-treated extracts. However, because we observe that RNA directly stimulates AurB we favor the possibility that the spindle assembly effects of RNase treatment are caused by loss of AurB activity. We found that the rescue was specific to MCAK inhibition and not a result of generally promoting microtubule polymerization, as ‘overexpression’ of recombinant EB1, which also promotes polymerization [59], had no effect on spindle assembly (Fig. 6C–D). This result is consistent with the observation that microtubule assembly factors are not fully redundant with each other [59]. The inability of MCAK inhibition to fully restore spindle assembly in RNase-treated extracts likely arises from pleiotropic effects of RNA on additional spindle assembly factors, such as Ran and Rae1, and from effects on other AurB targets. Nevertheless, the partial rescue of spindle assembly by MCAK inhibition demonstrates that the AurB-MCAK pathway is strongly affected by RNase treatment, and supports the idea that RNA activates AurB to promote spindle assembly.

## Discussion

Our results have identified an additional molecular pathway explaining the observation that RNA is required in a translation-independent manner for spindle assembly [35]. Here we have shown that RNA promotes maximal activation of the essential mitotic kinase AurB, and directly interacts with the CPC through multiple RNA-binding domains in the protein complex. RNase-treated extracts display several hallmarks of reduced AurB activity, including impaired spindle assembly, decreased Op18 hyperphosphorylation, and defects in MCAK localization at centromeres. The spindle morphology defects noted in RNase-treated extracts are largely mediated by MCAK, a microtubule-depolymerizing enzyme whose activity is downregulated by AurB-dependent phosphorylation [17], [21].

### RNA directly activates AurB kinase activity

AurB activity is highly regulated by multiple mechanisms, including its synthesis, degradation, subcellular localization, phosphorylation, and interaction with other proteins [11]. Several previous studies have suggested that RNA also regulates some aspect of AurB function. In somatic cells, mitotic chromosome spreads treated with RNase fail to localize Incenp and Survivin to metaphase chromosomes [37]. In addition, centromeres are regions of active transcription during mitosis [60], and low levels of transcription from the centromeric repeat sequences are required for proper centromere function [61]. Accordingly, AurB in mouse cells binds centromeric transcripts *in vivo* and is activated by them *in vitro* [36]. These studies suggest that repeat RNA is an active component of the centromere [62] and may serve to recruit and activate AurB there. While active transcription is important for centromere and kinetochore assembly in human cells, it is unlikely to impact centromeres in *Xenopus* eggs and early embryos, as several studies have shown that no transcription occurs in these cells [63]–[65]. Our study builds on these previous results, and represents the first genome-wide analysis of transcripts that associate with a centromere-localized factor. We found that AurB bound RNAs were very well correlated with transcripts that also localize to the spindle during mitosis. We did not identify any repetitive RNAs in our AurB IPs, but the unfinished nature of the *Xenopus* genome may have prevented identification of such sequences. Our results are consistent with these previous studies, as we have found that RNA is important for both the localization and activation of AurB, and suggest that AurB can bind to, and be activated by, a variety of RNA classes.

Our results have demonstrated that the interaction of the CPC with RNA *in vitro* occurs through RNA-binding sites in AurB and DasraA. Our *in vitro* experiments did not identify sequence-specific RNA binding in either of these domains, but it is possible that a more exhaustive examination of AurB-RNA interactions *in vitro* may identify a preferred binding site motif. A previous study of immunopurified mouse CPC hinted that the CPC may be a sequence-specific RNA binding complex as it was activated more effectively by minor satellite RNA compared to β-tubulin mRNA[36]. In agreement with this result, we found that the interaction of the CPC with RNA was not mediated through electrostatic interactions, as the CPC bound with much higher affinity to RNA compared to dsDNA. Crosslinking and Immunoprecipitation (CLIP) [66] experiments performed in live cells will be necessary to identify the RNA sites preferentially bound by the CPC *in vivo.*

In addition to defining RNA elements that stimulate AurB activity, it will be important to identify the domains and residues within AurB and Dasra/Borealin that are important for interacting with RNA. None of the members of the CPC contains a canonical RNA-binding domain, which is consistent with recent high-throughput approaches that find many proteins without annotated RNA-binding domains bind directly to mRNA [67], [68]. Understanding the domains of the CPC that interact directly with RNA will provide insight into the mechanism of kinase activation by RNA, and allow the creation of RNA-binding mutations that will facilitate testing the role of RNA-binding by the CPC *in vivo.*

Our work has shown that RNase treatment of egg extract leads to defects in spindle assembly and AurB activation. While the results of RNase treatment of egg extract, and the partial rescue of spindle assembly by MCAK inhibition, are consistent with a role for RNA in the activation of AurB, this treatment is likely to cause pleiotropic phenotypes, some of which are not related to inactivation of AurB. Therefore, specific point mutations of the CPC that prevent RNA binding will more precisely define the role of RNA in activating AurB and regulating spindle assembly during mitosis.

Our work expands the repertoire of proteins that are regulated by RNA binding [69], [70]. Many such proteins described to date are metabolic enzymes, e.g. aconitase [71] and GAPDH [72], and are typically inactivated by RNA binding (reviewed in [73]). Our work uncovers a role for RNA in stimulating enzymatic activity, suggesting that RNA can affect protein function in various ways.

## Materials and Methods

### Xenopus extracts, spindle assembly reactions

Egg extracts and spindles were prepared as described [74]. RNase treatment of extract was carried out by pre-incubating extract for 1 hr at 20°C with RNaseA TypeX IIA (Sigma) at a final concentration of 0.1 mg/ml (nearly identical results were obtained using RNase from Worthington or the combination of S-peptide and S-protein; not shown). The translation inhibitor puromycin was added at a final concentration of 0.1 mg/ml to extract for 30 min at 20°C. For cycled spindle assembly, extract containing sperm nuclei at a concentration of 500 nuclei/µl was cycled into interphase for 90 min with 0.6 mM CaCl_2_ [74]. Where indicated, Cy5-dUTP was added to cycling reactions at 2 µM. One volume of interphase extract was arrested with 2 volumes of extract treated with or without RNase as described. RNase concentration was adjusted to be 0.1 mg/ml throughout the experiment. Nocodazole was added at a final concentration of 10 µM where indicated. Rhodamine-labeled tubulin (Cytoskeleton, Inc.) was added at 10–20 µg/ml, GFP-AurB was added at 500 nM, and MBP-CyclinB Δ90 was added at 1 µM, and EB1-GFP was added at 750 nM, where indicated. Spindle reactions were diluted in 1x BrB80+30% glycerol+0.5% TritonX-100 and spun onto coverslips as described [74]. Coverslips were fixed in methanol at −20°C for 10 min. For immunofluorescence analysis and GFP-AurB quantitation, dilution buffer contained additional 4% paraformaldehyde, and methanol fixation was shortened to 2–3 min.

All images were obtained with an Olympus BX61 microscope equipped with a charge-coupled device camera (ORCA, Hamamatsu). Fixed images were acquired with the spinning disk confocal setting.

### Antibodies

Mouse αphospho-H3 (ser10) (Millipore) was used at 1/5000 for western blot. Rabbit αCenpE was a gift of Don Cleveland [75] and used at 1/500 for immunofluorescence and detected with αRabbit-Cy3 (Jackson Immunochemicals) at 1/1000. Sheep αMCAK antibody was a gift of Claire Walczak [18] and used at 2 µg/ml for immunofluorescence and detected with αSheep-Alexa 488 (Jackson Immunochemicals) at 1/1000. Mouse DM1a (anti-tubulin) was used at 1/5000 for western blot. Rabbit αAurB antibodies were generated by Covance against full-length denatured protein, and used at 0.1 µg/ml for western blot (see below for immunoprecipitations). Rabbit αRNaseA antibody was generated by affinity purifying serum raised against an S-peptide tagged protein with RNaseA coupled to CNBr-activated beads (Amersham), and used at 1 µg/ml for western blot. Rabbit αGFP antibodies were generated by Covance against a GFP-tagged protein, and affinity purified against recombinant GFP. Antibodies were used at a final concentration of 0.1 µg/ml for western blot (see below for immunoprecipitation). Alternatively, 40 µl of GFP-Trap beads (Allele Biotech) were used for GFP immunoprecipitations. Control, non-specific IgG antibodies (Jackson Immunochemicals) were used at equivalent concentrations as specific antibodies. Rabbit αOp18 (a gift from Rebecca Heald) was used at 0.08 µg/ml for western blot. Rabbit αDasraA and αSurvivin were gifts from Hironori Funabiki and Ming Tsai and used at 1/10,000 and 1/3000, respectively, for western blot. All western blots were detected with HRP-conjugated secondary antibodies from Jackson Immunochemicals at 1/10,000.

### αMCAK immunofluorescence

Nuclei were cycled through interphase in the presence or absence of Cy5-dUTP as described above. Unlabeled and Cy5-labeled interphase reactions were re-arrested in mitosis with control or RNase-treated extract, respectively, each containing rhodamine-labeled tubulin and 10 µM nocodazole, as described above. After 1 hr., reactions were fixed with paraformaldehyde as described above, combined, and spun onto a single coverslip. The coverslip was then processed for αMCAK immunofluorescence using Alexa488-labeled secondary antibody. Pairs of control and RNase-treated nuclei (distinguishable by the absence or presence, respectively, of Cy5 label in the chromosomes) lying in the same field of view were selected for analysis. Kinetochores were selected by manual thresholding based on the Alexa488 signal on control nuclei, and the integrated intensity of the remaining foci on each nucleus was computed using Metamorph.

### Luciferase Assays

Renilla Luciferase DNA (Promega) without (pMB 341) or with (pMB 725, a gift of Joe Avruch) an IRES sequence was transcribed *in vitro*. Capping reactions were conducted with the ScriptCap m7G capping system as described (CellScript). IRES-containing RNA was not capped. RNA was incubated at 10 nM in extracts at 20°C for 1 hr., and luciferase activity measured using the Dual-Glo Lucifearse Assay System (Promega).

### Kinase assays

Kinase assays were performed as described [39]. Briefly, AurB was immunoprecipitated from 30 µl control or RNase-treated extracts pre-incubated with sperm nuclei for 1 hr. Immunoprecipitations were conducted using 0.5 µg αAurB antibodies bound to Protein A dynabeads (Invitrogen). Kinase assays with recombinant GFP-AurB were performed by incubating the recombinant protein at a concentration of 500 nM with sperm nuclei in extracts for 1 hr., followed by immunoprecipitation with 5 µg α GFP antibody as described above. Beads were washed in 1 ml XB without calcium or sucrose (10 mM HEPES, 100 mM KCl, 1 mM MgCl_2 _pH7.7) plus 300 mM NaCl and 0.1% TritonX-100, and collected on a magnet. Beads were subsequently washed in 1 ml PBS with or without RNaseA at 0.01 mg/ml and rotated at room temperature for 10 min. A second wash with RNaseA was conducted without incubation, then beads were washed 3 more times with XB+ NaCl+ TritonX-100 as before. Beads were washed once with kinase buffer (20 mM Tris pH 7.5, 1 mM MgCl_2_, 25 mM KCl, 1 mM DTT, 40 µg/ml BSA). Kinase reactions with MCAK substrate contained 0.1 mM ATP, 5 µM GFP-MCAK-NT, 0.5 µCi g^32^P-ATP, and AurB bound to 0.1 µg αAurB antibody on beads in 20 µl kinase buffer. For kinase assay rescues, RNA was prepared from separate extracts and corresponding AurB immunoprecipitations using the RNeasy kit (Qiagen) as described below. Reactions were incubated for 15 min at room temperature. Beads were collected on a magnet and the supernatant was saved in final 1x SDS loading dye. Samples were run on 10% PAGE, the gel was then dried and analyzed for ^32^P incorporation using the PhosphorImager system. Reactions with histone H3 substrate included 2 mg/ml histones (Type VIII-S) instead of GFP-MCAK-NT, and were detected by western blot.

### *In vitro* kinase assays

Kinase assays were performed as described [52]. Protein complexes were added at a concentration of 600 nM to 5 µl λ phosphatase reactions containing 100 U λ phosphatase. Phosphatase reactions were incubated at 25°C (CPC) or 30°C (AurB-Incenp) for 30 min. Reactions were diluted to a final volume of 10 µl containing 0.1 mM ATP, 0.25 µCi g^32^P-ATP, 0.5x kinase buffer (see above), and 1 mM sodium vanadate to inhibit the phosphatase. 500 nM of RNA or double-stranded DNA was added as indicated. Reactions were incubated at room temperature for 15 minutes, then stopped by the addition of SDS loading dye. Samples were analyzed as described above.

### RNA sequencing

AurB was immunoprecipitated from 100 µl of extract treated with puromycin for 30 min. Immunoprecipitation was conducted on ice for 1 hr with 80 µg αAurB antibody. Beads were washed five times with 1 ml PBS+1% TritonX-100. RNA was isolated according to the RNeasy kit “RNA clean up” protocol (Qiagen). RNA libraries were prepared for sequencing using the TruSeq RNA Sample Preparation v2 kit (Illumina) according to manufacturer’s instructions. Four libraries were barcoded and sequenced per lane on an Illumina HiSeq machine. Comparison of enrichment of AurB associated RNAs was performed by sequencing three biological replicates of RNA from AurB immunoprecipitations and from matched total extracts. Comparison of changes in AurB- bound RNAs upon changes in cell cycle state (mitotic vs interphase extracts) was performed from a single extract by comparing the enrichment of RNA in AurB immunoprecipitations vs total extract in each phase. Sequencing reads were aligned to the *Xenopus laevis* Unigene transcript database using Bowtie [76] allowing for 1 mismatch per sequence. Reads per transcript and reads per Kb per million mapped were calculated using a custom Perl script. Graphs and statistical analysis of sequencing data were performed using R.

Spindle-associated RNAs were obtained by diluting cycled spindles in 1x BrB80+30% glycerol +0.5% TritonX-100 and pelleting through a 10 ml 1x BrB80+60% glycerol cushion as described [22]. RNA was isolated by Trizol (Life Technologies) extraction. Strand-specific Illumina libraries were prepared using the Illumina Tru-Seq kit and aligned to the *X. laevis* Unigene database as described for AurB-associated RNAs. To perform correlation analysis between AurB-bound and spindle-bound RNAs, we examined only transcripts that had a total of 100 reads in each sample.

RNA sequences associated with this study have been deposited into the NIH SRA under accession numbers (Bioproject: PRJNA191571 and PRJNA247381).

Motif analysis was performed by obtaining the sequences for all RNAs enriched in AurB IPs greater than two standard deviations from the mean, and filtered against a negative-control set of sequences derived from transcripts that were under-represented in AurB immunoprecipitations. The negative control set was four times larger than the set of AurB-enriched RNAs in terms of total nucleotide length. Prior to motif or base composition analysis, poly-A tails were removed from sequences. These sequences were used as input for the MEME motif discovery software (http://meme.sdsc.edu/meme/cgi-bin/meme.cgi) using the discriminatory motif discovery mode.

Gene Ontology analysis was performed by using BlastX to search the *X. laevis* Unigene transcripts against the human Uniprot protein sequences. Human Uniprot accession numbers for AurB-enriched transcripts and all expressed transcripts were used as input for GO analysis using DAVID[40].

Binding of endogenous transcripts to AurB was verified by isolating AurB-bound RNA as described above, and converting to cDNA with random hexamer primers using Superscript III reverse transcriptase (Life Technologies). Individual transcripts were quantified using the iQ SYBR Green system (Bio-Rad).

### In-extract RNA binding assays

A 318 bp fragment of Xl. 84202 was amplified from *Xenopus laevis* cDNA with primers oMB 1668 (CAGGACTTGGTTGAGACAAAATG) and oMB 1669 (GAGCATCCAATTCTTCATCACC), and a 320 bp fragment of Xl. 19006 was amplified similarly using primers oMB 1644 (CAAATTGATAAAGAGACAGATG) and oMB 1645 (TGCTCTCCAAGATGCTTGTCC). Both fragments were cloned into pCR2.1 TOPO (Invitrogen) (pMB 731 and 732, respectively). A 41 nt region identified by MEME motif analysis in pMB 732, consisting of bases GGCAAAATCAGATCACAAATCTGAAAGAGAAAAAGTAGAAA was mutated to ATGGGGCTGAGCTGTGGGCTCAGGGAGAGGGGGACGAGGG (pMB 733). DNA templates were amplified with T7 and M13 Reverse primers and used for *in vitro* transcription with T7 RNA polymerase. The resulting RNAs were capped as described for luciferase assays, and added to extracts together at a final concentration of 1 nM each. *In vitro* transcribed RNAs were incubated in extracts for 30 min., followed by treatment with puromycin for 30 min. RNA bound to AurB was isolated as described for RNA sequencing, reverse transcribed with random hexamer primers, and quantified by qPCR as described above using a plasmid-specific forward primer oMB 681 (GGGCCCTCTAGATGCATGCTC) and the following reverse primers: oMB 1669 (GAGCATCCAATTCTTCATCACC) for Xl. 84202, oMB 1810 (CTCTTTCAGATTTGTGATCTG) for Xl. 19006 WT, or oMB 1811 (TCTCCCTGAGCCCACAGCTCAG) for Xl. 19006 mutant.

### Protein expression and purification

GFP-3xHA-AurB was made by cloning full-length AurB DNA with an N-terminal 3xHA tag into a GFP-pET30a vector (pMB 599). GFP-MCAK-NT was made by cloning DNA encoding amino acids 2–149 of MCAK into a GFP-pET30a vector (pMB 730). Plasmids encoding AurB/Incenp and Survivin/DasraA have been previously described [52]. Survivin (with or without DasraA) was expressed by cloning the appropriate ORFs into pET30a (pMB 765, 766, respectively). Expression constructs were transformed into BL21 Rosetta (Promega). Cultures were grown to an OD 0.4–0.6 and IPTG was added to 0.1 mM, and cells were grown overnight at 16°C. Cells were collected by centrifugation and resuspended in the following buffers: AuroraB/Incenp, AuroraB/Incenp/Survivin/DasraA in PBS with 10 mM Imadazole and Protease Inhibitors (LPC and PMSF); Survivin, Survivin/DasraA, GFP-Aurora-B, His-3XHA-Aurora-B-N in PBS+350 mM NaCl+10 mM Imadazole and Protease Inhibitors. Cells were resuspended in 20 mls of buffer per 1 L of initial culture. Cells were lysed by a single passage through a French Press (1200 Psi) and 1 µL of Benzonase was added. Lysates were cleared by centrifugation 25,000×g for 30 min. Proteins were purified using Ni-NTA (Qiagen) and washed with ∼50–100 column volumes of lysis buffer. Proteins were eluted using lysis buffer+500 mM Imadazole. Eluted proteins were immediately applied to a gel filtration column (Superdex 75 for: Survivin, Survivin/DasraA, 3XHA-Aurora-B-N), (Superdex 200 for GFP-Aurora-B, Aurora-B/Incenp, or CPC). Peak fractions were collected, quantifed by Bradford, and stored at −80°C. CyclinB Δ90 was expressed in DH10 cells as a fusion to MBP, and purified over a maltose column.

### RNA EMSA

RNAs were produced by *in vitro* transcription using PCR products as a template and T7 RNA polymerase. *In vitro* transcripts were labeled with Cy5-CTP (ratio of incorporation: 1∶20). RNAs were purified by LiCl precipitation, diluted to 100 nM and stored at −80°C.

Final EMSA reactions contained: 20 mM Hepes (pH 7.8), 100 mM NaCl, 2 mM MgCl2, 4% glycerol, 1 mM beta-mercaptoethanol, 0.1 mg/ml yeast tRNA, 1 nM Cy5-labeled transcript and varying amounts of recombinant protein. 20 µL reactions were incubated at room temperature for 30 min and separated on 4% native acrylamide gels (prepared with 19∶1 acrylamide: bis-acrylamide). Gels were electrophoresed for 1 hr at 110 Volts and scanned using a Typhoon Imager (GE).

Percentage of RNA bound by each protein was calculated using ImageJ. Apparent dissociation constants were calculated from each lane using the equation:

K_d_  =  (([Protein]−[complex])*([RNA]−[complex]))/[complex]. Each titration was performed a minimum of two independent times and the reported K_d_ is an average of all lanes from two trials. K_d_ plots were produced using R.

### Ethics Statement

All animal work was performed according to standards of animals care and approved by MGH IACUC (OLAW Assurance #: A3596-01). All animal work performed in this study was approved by the Massachusetts General Hospital Subcommittee on Research Animal Care. Frogs were housed in Aquatic Habitats recirculating water housing systems. Water was maintained at a conductivity of ∼1800 µS and a pH between 7.5–8. Animals were fed frog brittle (Nasco). Frogs were provided with PVC tubes and plastic lily pads as enrichment. Frogs are handled minimally and all injections are performed using the best possible practices to minimize distress during handling.

## Supporting Information

Figure S1 **AurB kinase activity requires RNA in a translation-independent manner.** **A)** Hyperphosphorylation of Op18 in control or RNase-treated extracts containing phosphatase inhibitors, and sperm nuclei at a concentration of 5000/µl. Arrow indicates AurB-dependent hyperphosphorylated form of Op18. Tubulin is shown as a loading control. **B)** Indicated volumes of total egg extract were probed by western blot using custom αAurB antibody. Arrow indicates AurB. **C)** Phosphorylation of GFP-PP7 (non-specific protein) or GFP-MCAK-NT by AurB *in vitro*. **D)** Phosphorylation of histone H3 *in vitro* by AurB isolated from control or RNase-treated extracts incubated with sperm nuclei, and washed in the presence or absence of RNase. AurB is shown as a loading control. **E)** Same as (D), using recombinant AurB added to extracts and immunoprecipitated with anti-GFP antibody. D-E) Data are representative of experiments performed at least in triplicate. **F)** Inhibition of cap-dependent and IRES-dependent translation by puromycin. Luciferase RNA containing either a 5′ cap or the IRES sequence from HIV-1 was translated in extracts, and protein levels were monitored after 1 hour of incubation. n = 3 extracts. Error bars represent SEM. Luciferase RNA levels were comparable in control and puromycin-treated extracts during the course of the assay as assessed by RT-PCR. Total RNA is shown as a loading control. The decrease in IRES RNA during the experiment results from degradation due to an absence of a 5′ cap; note that degradation is unaffected by puromycin. **G)** Phosphorylation of MCAK *in vitro* by AurB isolated from mitotic extracts in the presence or absence of the translation inhibitor puromycin. Activity of AurB from interphase extract is also shown. All extracts were incubated with sperm nuclei prior to AurB isolation. MCAK substrate and AurB amounts are shown as loading controls. Data are representative of experiments performed at least in triplicate. **H)** Mitotic extract was incubated with sperm nuclei for 1 hr., and subjected to immunoprecipitation with αAurB antibodies. RNA was isolated from total extract prior to immunoprecipitation (input), or from the immunoprecipitation (AurB IP). The resulting RNA pools were added to reactions shown in Fig. 1 as indicated. **I)** Detection of RNaseA by western blot in input and AurB immunoprecipitations. 0.5 µl of control extract, or 0.05, 0.1, 0.2, 0.5 µl of RNase-treated extract (containing 5, 10, 20, or 50 ng RNaseA, respectively) were run in parallel with control or RNase-treated AurB immunoprecipitations. **J)** RNA added to kinase reactions pre-treated with RNase is stable. Total RNA was incubated with RNasin (0.8 U/µl) and with or without AurB beads treated with RNase as indicated. Data are representative of experiments performed at least in duplicate. **K)** Phosphorylation of MCAK *in vitro* by AurB isolated from control extract containing sperm nuclei. Each indicated RNA type was added at a concentration of 1.25 µg/ml. Data are representative of experiments performed at least in duplicate.
(TIF)

Figure S2 **Binding of CPC complex members to RNA** ***in vitro*** **.** **A)** Purified proteins used in gel shift assays. **B)** Purified CPC was blotted using myc antibody, which recognizes the myc epitope present on the C-terminus of Incenp. Western blot demonstrates the presence of many Incenp degradation products that co-purify with the CPC. Gel shift assays to assess binding of **C)** GFP-3xHA-AurB, **D)** Survivin, **E)** Survivin/DasraA, or **F)** AurB/Incenp to RNA fragments derived from Xl. 19006, Xl. 19006 lacking the poly-A motif defined in Fig. 2, Xl. 84202, or DNA encoding Xl. 19006.
(TIF)

Figure S3 **Binding of CPC complexes to RNA** ***in vitro*** **(see** **Fig. 3** **).** Quantificaiton of RNA binding of **A)** GFP-AurB, **B)** Survivin, **C)** Survivin/DasraA, **D)** AurB/Incenp, or **E)** full CPC to Xl. 19006, Xl. 19006 mutant, or Xl. 84202 RNAs, or Xl. 19006 DNA. Data from 2–3 independent EMSA experiments from each protein-nucleic acid combination are shown. Best-fit curve for each combination is plotted in blue. Independent experiments are indicated by circle and triangle plotting symbols.
(TIF)

Figure S4 **RNA stimulates AurB** ***in vitro*** **and is required for proper spindle morphology in extracts.** **A)** Phosphorylation of AurB and Incenp *in vitro* by purified, full CPC in the presence or absence of Xl. 84202 or Xl. 19006 transcripts. **B)** Quantitation of spindle lengths from Fig. 6A. (n = 3 extracts, 20–25 spindles per extract per condition, p<0.01 by paired t-test of mean values from each extract). Error bars represent SEM.
(TIF)

Table S1 **Gene ontology analysis of AurB and spindle –enriched transcripts.** Transcripts enriched in AurB IP (Tab1), purified spindles (Tab2), and both (Tab3) were used as input for the DAVID Gene Ontology browser. Prior to analysis all *X. laevis* Unigene transcripts were converted into human Uniprot names using BlastX. Significantly enriched categories are presented.
(XLSX)

Table S2 **Correlations between two different, representative sequencing libraries.** Pearson correlation coefficients were calculated using RPKM per transcript from sequencing libraries derived from total RNA in 2 separate extracts, or from RNA co-immunoprecipitated with AurB from the corresponding extracts. In addition, the correlation of transcript enrichment in the AurB immunoprecipitation (Aurora-B IP(rpkm)/Total extract(rpkm)) was calculated with respect to the relative enrichment of each transcript on purified spindles, and with the base composition of each transcript (% of each base).
(DOCX)

Table S3 Sequencing reads aligned to *X. laeivs* Unigene sequences from two AurB IP and Total extract pairs, and from two purified spindle and Total extract pairs. Data are presented as raw read counts and normalized RPKM values for each library.
(TXT)

## References

[1] Mitchison T, Kirschner M (1984) Microtubule assembly nucleated by isolated centrosomes. Nature 312: 232–237. 6504137 10.1038/312232a0

[2] Heald R, Tournebize R, Blank T, Sandaltzopoulos R, Becker P, et al (1996) Self-organization of microtubules into bipolar spindles around artificial chromosomes in Xenopus egg extracts. Nature 382: 420–425. 8684481 10.1038/382420a0

[3] Karsenti E, Newport J, Kirschner M (1984) Respective roles of centrosomes and chromatin in the conversion of microtubule arrays from interphase to metaphase. J Cell Biol 99: 47s–54s. 6235234 10.1083/jcb.99.1.47s PMC2275591

[4] Gadde S, Heald R (2004) Mechanisms and molecules of the mitotic spindle. Curr Biol 14: R797–805. 15380094 10.1016/j.cub.2004.09.021

[5] Nachury MV, Maresca TJ, Salmon WC, Waterman-Storer CM, Heald R, et al (2001) Importin beta is a mitotic target of the small GTPase Ran in spindle assembly. Cell 104: 95–106. 11163243 10.1016/s0092-8674(01)00194-5

[6] Wiese C, Wilde A, Moore MS, Adam SA, Merdes A, et al (2001) Role of importin-beta in coupling Ran to downstream targets in microtubule assembly. Science 291: 653–656. 11229403 10.1126/science.1057661

[7] Gruss OJ, Carazo-Salas RE, Schatz CA, Guarguaglini G, Kast J, et al (2001) Ran induces spindle assembly by reversing the inhibitory effect of importin alpha on TPX2 activity. Cell 104: 83–93. 11163242 10.1016/s0092-8674(01)00193-3

[8] Koffa MD, Casanova CM, Santarella R, Kocher T, Wilm M, et al (2006) HURP is part of a Ran-dependent complex involved in spindle formation. Curr Biol 16: 743–754. 16631581 10.1016/j.cub.2006.03.056

[9] Sillje HH, Nagel S, Korner R, Nigg EA (2006) HURP is a Ran-importin beta-regulated protein that stabilizes kinetochore microtubules in the vicinity of chromosomes. Curr Biol 16: 731–742. 16631580 10.1016/j.cub.2006.02.070

[10] Ribbeck K, Groen AC, Santarella R, Bohnsack MT, Raemaekers T, et al (2006) NuSAP, a mitotic RanGTP target that stabilizes and cross-links microtubules. Mol Biol Cell 17: 2646–2660. 16571672 10.1091/mbc.E05-12-1178 PMC1474800

[11] Carmena M, Wheelock M, Funabiki H, Earnshaw WC (2012) The chromosomal passenger complex (CPC): from easy rider to the godfather of mitosis. Nat Rev Mol Cell Biol 13: 789–803. 23175282 10.1038/nrm3474 PMC3729939

[12] Earnshaw WC, Bernat RL (1991) Chromosomal passengers: toward an integrated view of mitosis. Chromosoma 100: 139–146. 2040201 10.1007/BF00337241

[13] Ruchaud S, Carmena M, Earnshaw WC (2007) Chromosomal passengers: conducting cell division. Nat Rev Mol Cell Biol 8: 798–812. 17848966 10.1038/nrm2257

[14] Gadea BB, Ruderman JV (2006) Aurora B is required for mitotic chromatin-induced phosphorylation of Op18/Stathmin. Proc Natl Acad Sci U S A 103: 4493–4498. 16537398 10.1073/pnas.0600702103 PMC1401233

[15] Belmont LD, Mitchison TJ (1996) Identification of a protein that interacts with tubulin dimers and increases the catastrophe rate of microtubules. Cell 84: 623–631. 8598048 10.1016/s0092-8674(00)81037-5

[16] Andrews PD, Ovechkina Y, Morrice N, Wagenbach M, Duncan K, et al (2004) Aurora B regulates MCAK at the mitotic centromere. Dev Cell 6: 253–268. 14960279 10.1016/s1534-5807(04)00025-5

[17] Lan W, Zhang X, Kline-Smith SL, Rosasco SE, Barrett-Wilt GA, et al (2004) Aurora B phosphorylates centromeric MCAK and regulates its localization and microtubule depolymerization activity. Curr Biol 14: 273–286. 14972678 10.1016/j.cub.2004.01.055

[18] Desai A, Verma S, Mitchison TJ, Walczak CE (1999) Kin I kinesins are microtubule-destabilizing enzymes. Cell 96: 69–78. 9989498 10.1016/s0092-8674(00)80960-5

[19] Tournebize R, Popov A, Kinoshita K, Ashford AJ, Rybina S, et al (2000) Control of microtubule dynamics by the antagonistic activities of XMAP215 and XKCM1 in Xenopus egg extracts. Nat Cell Biol 2: 13–19. 10620801 10.1038/71330

[20] Walczak CE, Mitchison TJ, Desai A (1996) XKCM1: a Xenopus kinesin-related protein that regulates microtubule dynamics during mitotic spindle assembly. Cell 84: 37–47. 8548824 10.1016/s0092-8674(00)80991-5

[21] Ohi R, Sapra T, Howard J, Mitchison TJ (2004) Differentiation of cytoplasmic and meiotic spindle assembly MCAK functions by Aurora B-dependent phosphorylation. Mol Biol Cell 15: 2895–2906. 15064354 10.1091/mbc.E04-02-0082 PMC420112

[22] Blower MD, Feric E, Weis K, Heald R (2007) Genome-wide analysis demonstrates conserved localization of messenger RNAs to mitotic microtubules. J Cell Biol 179: 1365–1373. 18166649 10.1083/jcb.200705163 PMC2373496

[23] Eliscovich C, Peset I, Vernos I, Mendez R (2008) Spindle-localized CPE-mediated translation controls meiotic chromosome segregation. Nat Cell Biol 10: 858–865. 18536713 10.1038/ncb1746

[24] Groisman I, Huang YS, Mendez R, Cao Q, Theurkauf W, et al (2000) CPEB, maskin, and cyclin B1 mRNA at the mitotic apparatus: implications for local translational control of cell division. Cell 103: 435–447. 11081630 10.1016/s0092-8674(00)00135-5

[25] Sharp JA, Plant JJ, Ohsumi TK, Borowsky M, Blower MD (2011) Functional analysis of the microtubule-interacting transcriptome. Mol Biol Cell 22: 4312–4323. 21937723 10.1091/mbc.E11-07-0629 PMC3216657

[26] Kingsley EP, Chan XY, Duan Y, Lambert JD (2007) Widespread RNA segregation in a spiralian embryo. Evol Dev 9: 527–539. 17976050 10.1111/j.1525-142X.2007.00194.x

[27] Lambert JD, Nagy LM (2002) Asymmetric inheritance of centrosomally localized mRNAs during embryonic cleavages. Nature 420: 682–686. 12478296 10.1038/nature01241

[28] Lecuyer E, Yoshida H, Parthasarathy N, Alm C, Babak T, et al (2007) Global analysis of mRNA localization reveals a prominent role in organizing cellular architecture and function. Cell 131: 174–187. 17923096 10.1016/j.cell.2007.08.003

[29] Rabinowitz JS, Lambert JD (2010) Spiralian quartet developmental potential is regulated by specific localization elements that mediate asymmetric RNA segregation. Development 137: 4039–4049. 21041364 10.1242/dev.055269

[30] Raff JW, Whitfield WG, Glover DM (1990) Two distinct mechanisms localise cyclin B transcripts in syncytial Drosophila embryos. Development 110: 1249–1261. 2151612 10.1242/dev.110.4.1249

[31] Audhya A, Hyndman F, McLeod IX, Maddox AS, Yates JR 3rd, et al (2005) A complex containing the Sm protein CAR-1 and the RNA helicase CGH-1 is required for embryonic cytokinesis in Caenorhabditis elegans. J Cell Biol 171: 267–279. 16247027 10.1083/jcb.200506124 PMC2171198

[32] Goshima G, Wollman R, Goodwin SS, Zhang N, Scholey JM, et al (2007) Genes required for mitotic spindle assembly in Drosophila S2 cells. Science 316: 417–421. 17412918 10.1126/science.1141314 PMC2837481

[33] Hussain S, Benavente SB, Nascimento E, Dragoni I, Kurowski A, et al (2009) The nucleolar RNA methyltransferase Misu (NSun2) is required for mitotic spindle stability. J Cell Biol 186: 27–40. 19596847 10.1083/jcb.200810180 PMC2712989

[34] Squirrell JM, Eggers ZT, Luedke N, Saari B, Grimson A, et al (2006) CAR-1, a protein that localizes with the mRNA decapping component DCAP-1, is required for cytokinesis and ER organization in Caenorhabditis elegans embryos. Mol Biol Cell 17: 336–344. 16267265 10.1091/mbc.E05-09-0874 PMC1345671

[35] Blower MD, Nachury M, Heald R, Weis K (2005) A Rae1-containing ribonucleoprotein complex is required for mitotic spindle assembly. Cell 121: 223–234. 15851029 10.1016/j.cell.2005.02.016

[36] Ferri F, Bouzinba-Segard H, Velasco G, Hube F, Francastel C (2009) Non-coding murine centromeric transcripts associate with and potentiate Aurora B kinase. Nucleic Acids Res 37: 5071–5080. 19542185 10.1093/nar/gkp529 PMC2731909

[37] Wong LH, Brettingham-Moore KH, Chan L, Quach JM, Anderson MA, et al (2007) Centromere RNA is a key component for the assembly of nucleoproteins at the nucleolus and centromere. Genome Res 17: 1146–1160. 17623812 10.1101/gr.6022807 PMC1933521

[38] Hsu JY, Sun ZW, Li X, Reuben M, Tatchell K, et al (2000) Mitotic phosphorylation of histone H3 is governed by Ipl1/aurora kinase and Glc7/PP1 phosphatase in budding yeast and nematodes. Cell 102: 279–291. 10975519 10.1016/s0092-8674(00)00034-9

[39] Bolton MA, Lan W, Powers SE, McCleland ML, Kuang J, et al (2002) Aurora B kinase exists in a complex with survivin and INCENP and its kinase activity is stimulated by survivin binding and phosphorylation. Mol Biol Cell 13: 3064–3077. 12221116 10.1091/mbc.E02-02-0092 PMC124143

[40] Huang da W, Sherman BT, Lempicki RA (2009) Systematic and integrative analysis of large gene lists using DAVID bioinformatics resources. Nat Protoc 4: 44–57. 19131956 10.1038/nprot.2008.211

[41] Edwards NS, Murray AW (2005) Identification of xenopus CENP-A and an associated centromeric DNA repeat. Mol Biol Cell 16: 1800–1810. 15673610 10.1091/mbc.E04-09-0788 PMC1073662

[42] Bailey TL, Boden M, Buske FA, Frith M, Grant CE, et al (2009) MEME SUITE: tools for motif discovery and searching. Nucleic Acids Res 37: W202–208. 19458158 10.1093/nar/gkp335 PMC2703892

[43] Hafner M, Landthaler M, Burger L, Khorshid M, Hausser J, et al (2010) Transcriptome-wide identification of RNA-binding protein and microRNA target sites by PAR-CLIP. Cell 141: 129–141. 20371350 10.1016/j.cell.2010.03.009 PMC2861495

[44] Hafner M, Max KE, Bandaru P, Morozov P, Gerstberger S, et al (2013) Identification of mRNAs bound and regulated by human LIN28 proteins and molecular requirements for RNA recognition. RNA 19: 613–626. 23481595 10.1261/rna.036491.112 PMC3677277

[45] Ince-Dunn G, Okano HJ, Jensen KB, Park WY, Zhong R, et al (2012) Neuronal Elav-like (Hu) proteins regulate RNA splicing and abundance to control glutamate levels and neuronal excitability. Neuron 75: 1067–1080. 22998874 10.1016/j.neuron.2012.07.009 PMC3517991

[46] Licatalosi DD, Mele A, Fak JJ, Ule J, Kayikci M, et al (2008) HITS-CLIP yields genome-wide insights into brain alternative RNA processing. Nature 456: 464–469. 18978773 10.1038/nature07488 PMC2597294

[47] Zhao J, Ohsumi TK, Kung JT, Ogawa Y, Grau DJ, et al (2010) Genome-wide identification of polycomb-associated RNAs by RIP-seq. Mol Cell 40: 939–953. 21172659 10.1016/j.molcel.2010.12.011 PMC3021903

[48] Davidovich C, Zheng L, Goodrich KJ, Cech TR (2013) Promiscuous RNA binding by Polycomb repressive complex 2. Nat Struct Mol Biol 20: 1250–1257. 24077223 10.1038/nsmb.2679 PMC3823624

[49] Makino DL, Baumgartner M, Conti E (2013) Crystal structure of an RNA-bound 11-subunit eukaryotic exosome complex. Nature 495: 70–75. 23376952 10.1038/nature11870

[50] Klein UR, Nigg EA, Gruneberg U (2006) Centromere targeting of the chromosomal passenger complex requires a ternary subcomplex of Borealin, Survivin, and the N-terminal domain of INCENP. Mol Biol Cell 17: 2547–2558. 16571674 10.1091/mbc.E05-12-1133 PMC1474794

[51] Kelly AE, Sampath SC, Maniar TA, Woo EM, Chait BT, et al (2007) Chromosomal enrichment and activation of the aurora B pathway are coupled to spatially regulate spindle assembly. Dev Cell 12: 31–43. 17199039 10.1016/j.devcel.2006.11.001 PMC1892535

[52] Rosasco-Nitcher SE, Lan W, Khorasanizadeh S, Stukenberg PT (2008) Centromeric Aurora-B activation requires TD-60, microtubules, and substrate priming phosphorylation. Science 319: 469–472. 18218899 10.1126/science.1148980

[53] Tseng BS, Tan L, Kapoor TM, Funabiki H (2010) Dual detection of chromosomes and microtubules by the chromosomal passenger complex drives spindle assembly. Dev Cell 18: 903–912. 20627073 10.1016/j.devcel.2010.05.018 PMC2905387

[54] Zhang X, Lan W, Ems-McClung SC, Stukenberg PT, Walczak CE (2007) Aurora B phosphorylates multiple sites on mitotic centromere-associated kinesin to spatially and temporally regulate its function. Mol Biol Cell 18: 3264–3276. 17567953 10.1091/mbc.E07-01-0086 PMC1951741

[55] Knowlton AL, Lan W, Stukenberg PT (2006) Aurora B is enriched at merotelic attachment sites, where it regulates MCAK. Curr Biol 16: 1705–1710. 16950107 10.1016/j.cub.2006.07.057

[56] Cimini D (2007) Detection and correction of merotelic kinetochore orientation by Aurora B and its partners. Cell Cycle 6: 1558–1564. 17603301 10.4161/cc.6.13.4452

[57] Sampath SC, Ohi R, Leismann O, Salic A, Pozniakovski A, et al (2004) The chromosomal passenger complex is required for chromatin-induced microtubule stabilization and spindle assembly. Cell 118: 187–202. 15260989 10.1016/j.cell.2004.06.026

[58] Mitchison TJ, Maddox P, Gaetz J, Groen A, Shirasu M, et al (2005) Roles of polymerization dynamics, opposed motors, and a tensile element in governing the length of Xenopus extract meiotic spindles. Mol Biol Cell 16: 3064–3076. 15788560 10.1091/mbc.E05-02-0174 PMC1142448

[59] Groen AC, Maresca TJ, Gatlin JC, Salmon ED, Mitchison TJ (2009) Functional overlap of microtubule assembly factors in chromatin-promoted spindle assembly. Mol Biol Cell 20: 2766–2773. 19369413 10.1091/mbc.E09-01-0043 PMC2688555

[60] Chan FL, Marshall OJ, Saffery R, Kim BW, Earle E, et al (2012) Active transcription and essential role of RNA polymerase II at the centromere during mitosis. Proc Natl Acad Sci U S A 109: 1979–1984. 22308327 10.1073/pnas.1108705109 PMC3277563

[61] Nakano M, Cardinale S, Noskov VN, Gassmann R, Vagnarelli P, et al (2008) Inactivation of a human kinetochore by specific targeting of chromatin modifiers. Dev Cell 14: 507–522. 18410728 10.1016/j.devcel.2008.02.001 PMC2311382

[62] Gent JI, Dawe RK (2012) RNA as a structural and regulatory component of the centromere. Annu Rev Genet 46: 443–453. 22974300 10.1146/annurev-genet-110711-155419

[63] Newport J, Kirschner M (1982) A major developmental transition in early Xenopus embryos: II. Control of the onset of transcription. Cell 30: 687–696. 7139712 10.1016/0092-8674(82)90273-2

[64] Newport J, Kirschner M (1982) A major developmental transition in early Xenopus embryos: I. characterization and timing of cellular changes at the midblastula stage. Cell 30: 675–686. 6183003 10.1016/0092-8674(82)90272-0

[65] Tan MH, Au KF, Yablonovitch AL, Wills AE, Chuang J, et al (2013) RNA sequencing reveals a diverse and dynamic repertoire of the Xenopus tropicalis transcriptome over development. Genome Res 23: 201–216. 22960373 10.1101/gr.141424.112 PMC3530680

[66] Darnell RB (2010) HITS-CLIP: panoramic views of protein-RNA regulation in living cells. Wiley Interdiscip Rev RNA 1: 266–286. 21935890 10.1002/wrna.31 PMC3222227

[67] Baltz AG, Munschauer M, Schwanhausser B, Vasile A, Murakawa Y, et al (2012) The mRNA-bound proteome and its global occupancy profile on protein-coding transcripts. Mol Cell 46: 674–690. 22681889 10.1016/j.molcel.2012.05.021

[68] Castello A, Fischer B, Eichelbaum K, Horos R, Beckmann BM, et al (2012) Insights into RNA biology from an atlas of mammalian mRNA-binding proteins. Cell 149: 1393–1406. 22658674 10.1016/j.cell.2012.04.031

[69] Castello A, Horos R, Strein C, Fischer B, Eichelbaum K, et al (2013) System-wide identification of RNA-binding proteins by interactome capture. Nat Protoc 8: 491–500. 23411631 10.1038/nprot.2013.020

[70] Kwon SC, Yi H, Eichelbaum K, Fohr S, Fischer B, et al (2013) The RNA-binding protein repertoire of embryonic stem cells. Nat Struct Mol Biol 20: 1122–1130. 23912277 10.1038/nsmb.2638

[71] Kennedy MC, Mende-Mueller L, Blondin GA, Beinert H (1992) Purification and characterization of cytosolic aconitase from beef liver and its relationship to the iron-responsive element binding protein. Proc Natl Acad Sci U S A 89: 11730–11734. 1334546 10.1073/pnas.89.24.11730 PMC50630

[72] Nagy E, Rigby WF (1995) Glyceraldehyde-3-phosphate dehydrogenase selectively binds AU-rich RNA in the NAD(+)-binding region (Rossmann fold). J Biol Chem 270: 2755–2763. 7531693 10.1074/jbc.270.6.2755

[73] Ciesla J (2006) Metabolic enzymes that bind RNA: yet another level of cellular regulatory network? Acta Biochim Pol 53: 11–32. 16410835

[74] Hannak E, Heald R (2006) Investigating mitotic spindle assembly and function in vitro using Xenopus laevis egg extracts. Nat Protoc 1: 2305–2314. 17406472 10.1038/nprot.2006.396

[75] Kim Y, Heuser JE, Waterman CM, Cleveland DW (2008) CENP-E combines a slow, processive motor and a flexible coiled coil to produce an essential motile kinetochore tether. J Cell Biol 181: 411–419. 18443223 10.1083/jcb.200802189 PMC2364708

[76] Langmead B, Trapnell C, Pop M, Salzberg SL (2009) Ultrafast and memory-efficient alignment of short DNA sequences to the human genome. Genome Biol 10: R25. 19261174 10.1186/gb-2009-10-3-r25 PMC2690996

[77] Schneider TD, Stephens RM (1990) Sequence logos: a new way to display consensus sequences. Nucleic Acids Res 18: 6097–6100. 2172928 10.1093/nar/18.20.6097 PMC332411

