# Supplementary figures and images for: RNA Stimulates Aurora B Kinase Activity during Mitosis
Source: PLoS One. 2014 Jun 26;9(6):e100748. doi: 10.1371/journal.pone.0100748 (PMC4072698; doi:10.1371/journal.pone.0100748)

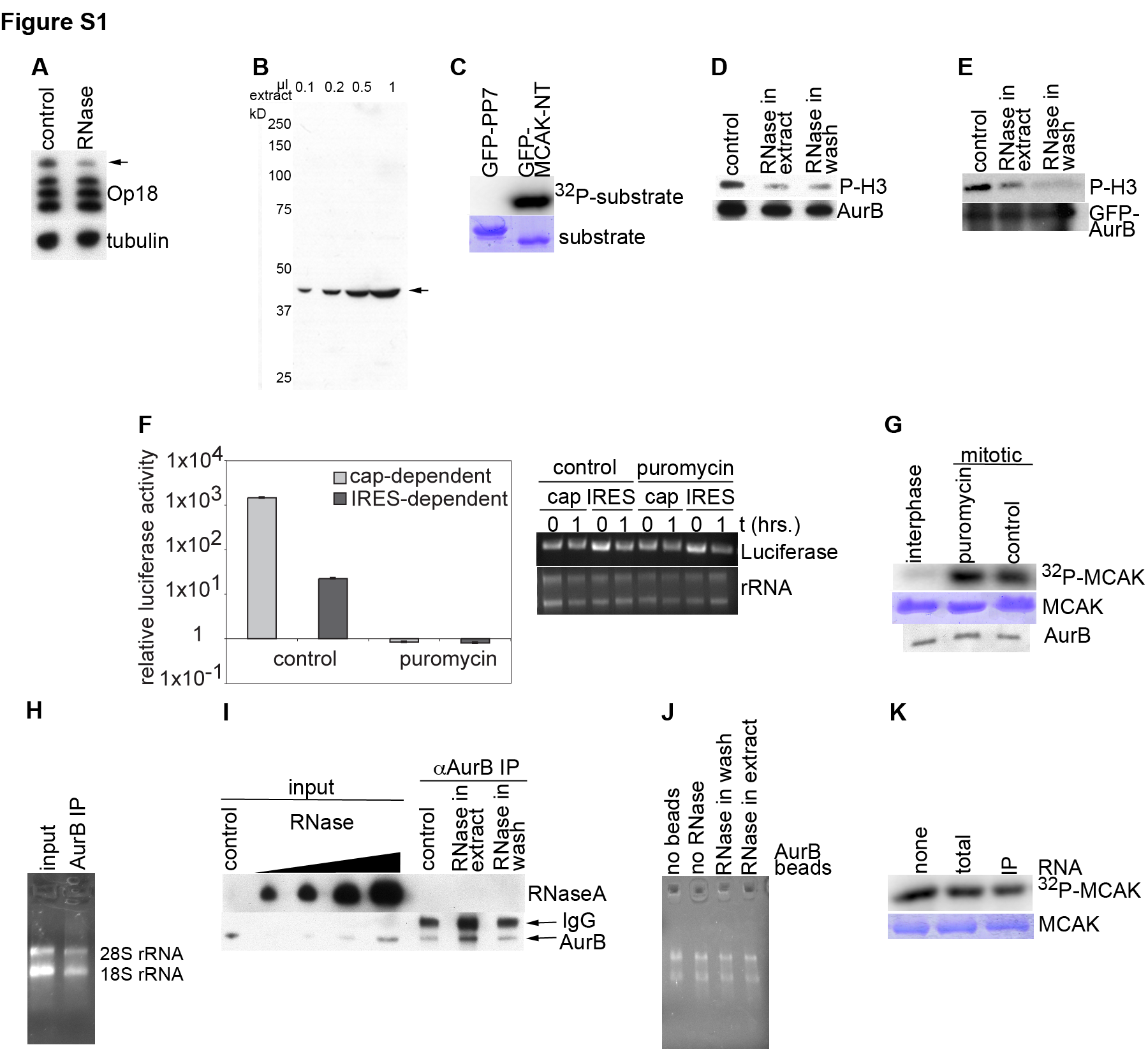

Supplement: Figure S1 — AurB kinase activity requires RNA in a translation-independent manner. A) Hyperphosphorylation of Op18 in control or RNase-treated extracts containing phosphatase inhibitors, and sperm nuclei at a concentration of 5000/µl. Arrow indicates AurB-dependent hyperphosphorylated form of Op18. Tubulin is shown as a loading control. B) Indicated volumes of total egg extract were probed by western blot using custom αAurB antibody. Arrow indicates AurB. C) Phosphorylation of GFP-PP7 (non-specific protein) or GFP-MCAK-NT by AurB in vitro. D) Phosphorylation of histone H3 in vitro by AurB isolated from control or RNase-treated extracts incubated with sperm nuclei, and washed in the presence or absence of RNase. AurB is shown as a loading control. E) Same as (D), using recombinant AurB added to extracts and immunoprecipitated with anti-GFP antibody. D-E) Data are representative of experiments performed at least in triplicate. F) Inhibition of cap-dependent and IRES-dependent translation by puromycin. Luciferase RNA containing either a 5′ cap or the IRES sequence from HIV-1 was translated in extracts, and protein levels were monitored after 1 hour of incubation. n = 3 extracts. Error bars represent SEM. Luciferase RNA levels were comparable in control and puromycin-treated extracts during the course of the assay as assessed by RT-PCR. Total RNA is shown as a loading control. The decrease in IRES RNA during the experiment results from degradation due to an absence of a 5′ cap; note that degradation is unaffected by puromycin. G) Phosphorylation of MCAK in vitro by AurB isolated from mitotic extracts in the presence or absence of the translation inhibitor puromycin. Activity of AurB from interphase extract is also shown. All extracts were incubated with sperm nuclei prior to AurB isolation. MCAK substrate and AurB amounts are shown as loading controls. Data are representative of experiments performed at least in triplicate. H) Mitotic extract was incubated with sperm nuclei for 1 h [file pone.0100748.s001.tif]

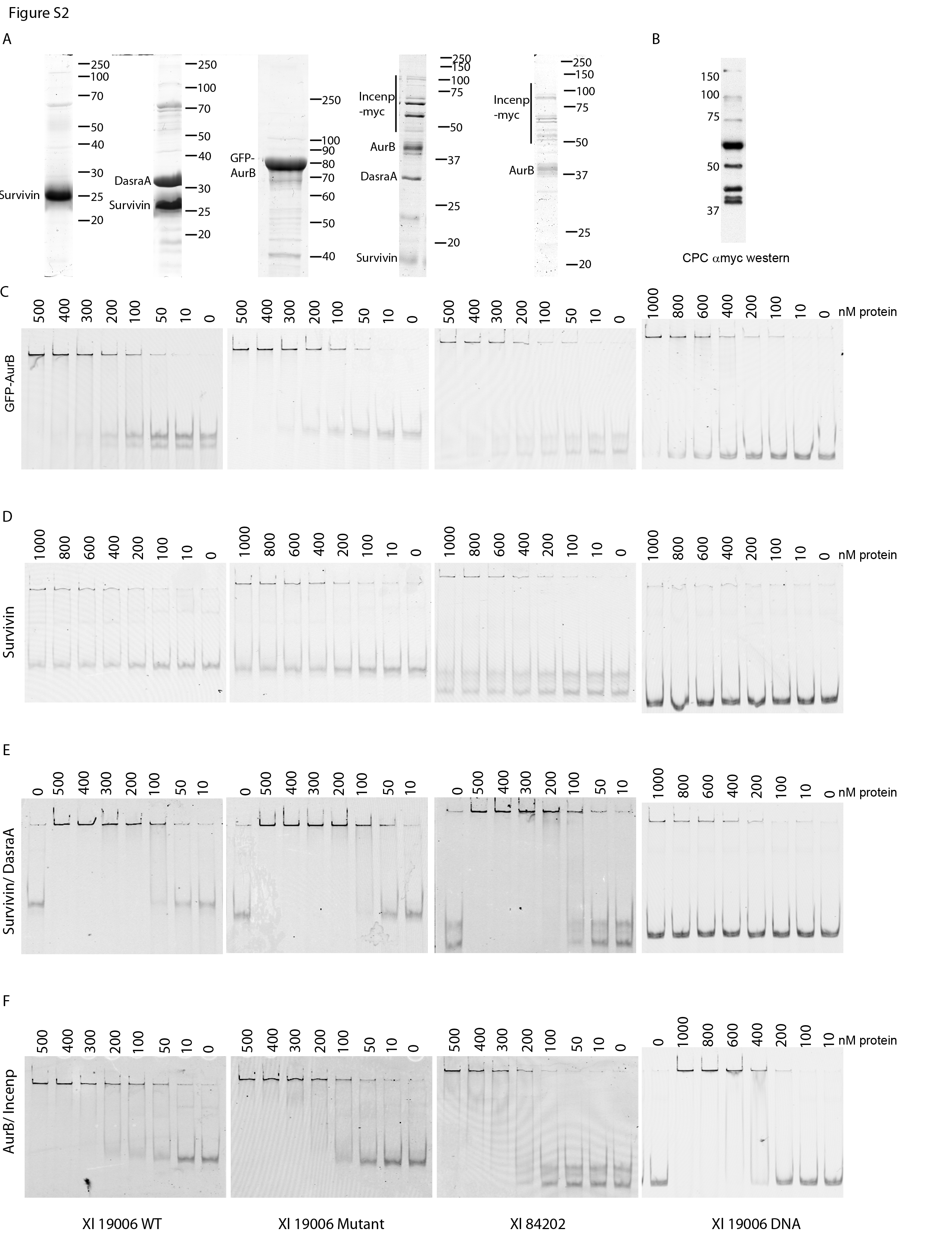

Supplement: Figure S2 — Binding of CPC complex members to RNA in vitro . A) Purified proteins used in gel shift assays. B) Purified CPC was blotted using myc antibody, which recognizes the myc epitope present on the C-terminus of Incenp. Western blot demonstrates the presence of many Incenp degradation products that co-purify with the CPC. Gel shift assays to assess binding of C) GFP-3xHA-AurB, D) Survivin, E) Survivin/DasraA, or F) AurB/Incenp to RNA fragments derived from Xl. 19006, Xl. 19006 lacking the poly-A motif defined in Fig. 2, Xl. 84202, or DNA encoding Xl. 19006. (TIF) [file pone.0100748.s002.tif]

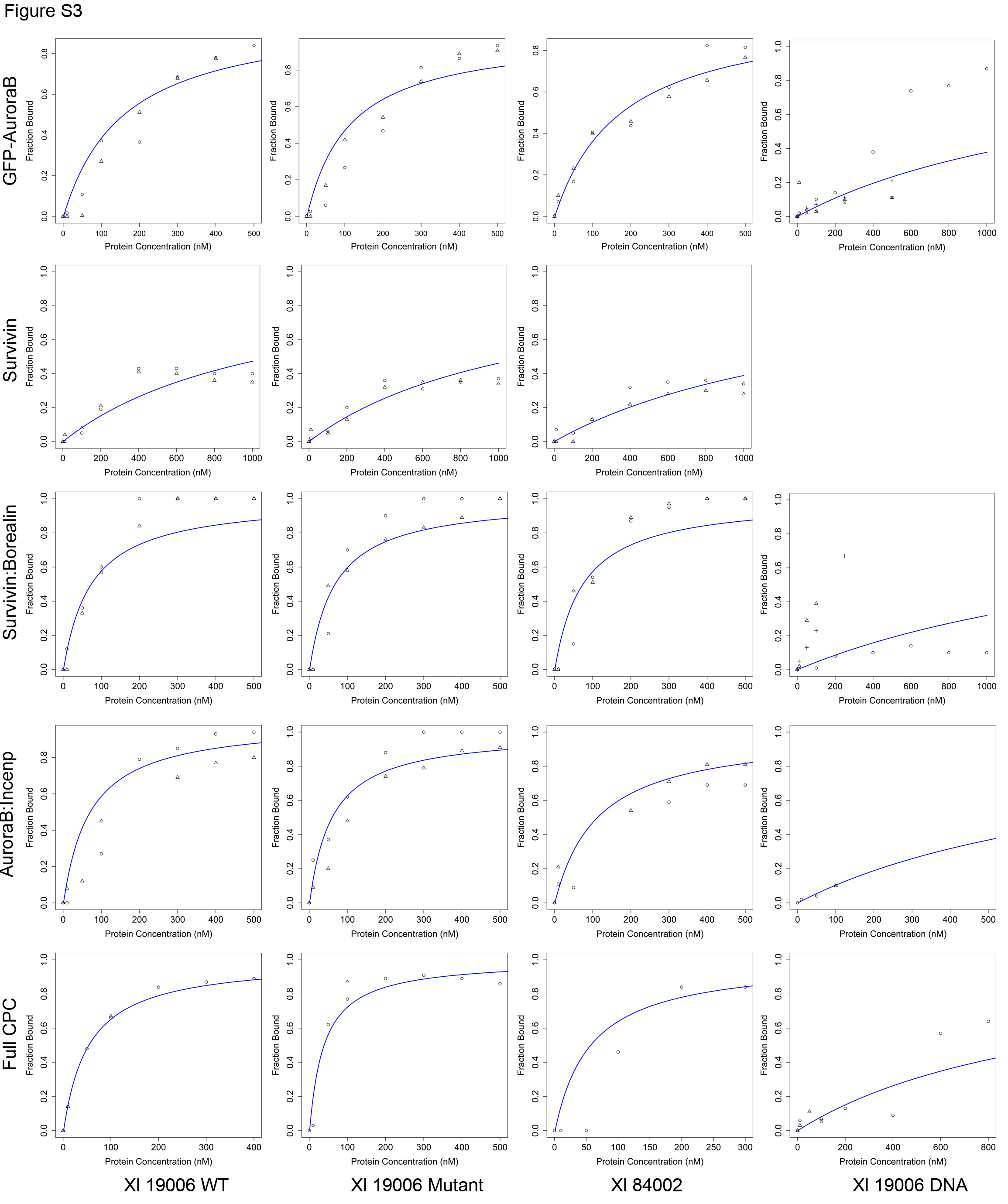

Supplement: Figure S3 — Binding of CPC complexes to RNA in vitro (see Fig. 3 ). Quantificaiton of RNA binding of A) GFP-AurB, B) Survivin, C) Survivin/DasraA, D) AurB/Incenp, or E) full CPC to Xl. 19006, Xl. 19006 mutant, or Xl. 84202 RNAs, or Xl. 19006 DNA. Data from 2–3 independent EMSA experiments from each protein-nucleic acid combination are shown. Best-fit curve for each combination is plotted in blue. Independent experiments are indicated by circle and triangle plotting symbols. (TIF) [file pone.0100748.s003.tif]

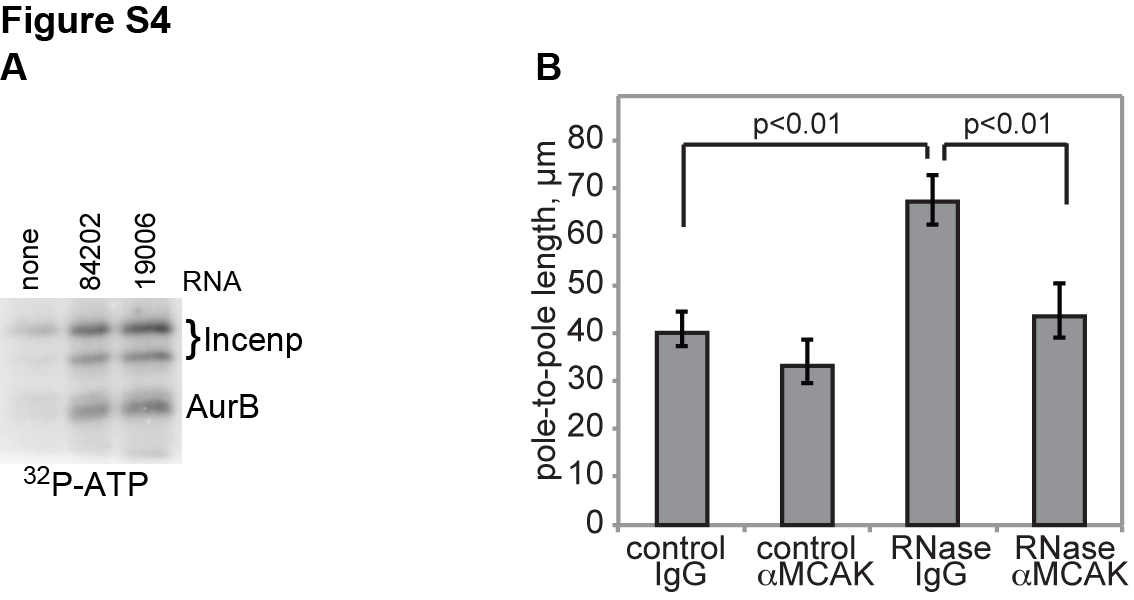

Supplement: Figure S4 — RNA stimulates AurB in vitro and is required for proper spindle morphology in extracts. A) Phosphorylation of AurB and Incenp in vitro by purified, full CPC in the presence or absence of Xl. 84202 or Xl. 19006 transcripts. B) Quantitation of spindle lengths from Fig. 6A. (n = 3 extracts, 20–25 spindles per extract per condition, p<0.01 by paired t-test of mean values from each extract). Error bars represent SEM. (TIF) [file pone.0100748.s004.tif]
