# Supplementary material for: RNA Stimulates Aurora B Kinase Activity during Mitosis
Source: PLoS One. 2014 Jun 26;9(6):e100748. doi: 10.1371/journal.pone.0100748 (PMC4072698; doi:10.1371/journal.pone.0100748)
Supplement: Table S2 — Correlations between two different, representative sequencing libraries. Pearson correlation coefficients were calculated using RPKM per transcript from sequencing libraries derived from total RNA in 2 separate extracts, or from RNA co-immunoprecipitated with AurB from the corresponding extracts. In addition, the correlation of transcript enrichment in the AurB immunoprecipitation (Aurora-B IP(rpkm)/Total extract(rpkm)) was calculated with respect to the relative enrichment of each transcript on purified spindles, and with the base composition of each transcript (% of each base). (DOCX) [file pone.0100748.s006.docx]

**Table S2.**

|  | **Total 1** | **Total 2** | **AurB IP 1** | **AurB IP 2** | **Spindle mRNAs** | **A** | **T** | **G** | **C** |
| --- | --- | --- | --- | --- | --- | --- | --- | --- | --- |
| **Total 1** | 1 |  |  |  |  |  |  |  |  |
| **Total 2** | 0.99 | 1 |  |  |  |  |  |  |  |
| **AurB IP 1** | 0.34 | 0.39 | 1 |  |  |  |  |  |  |
| **AurB IP 2** | 0.44 | 0.49 | 0.96 | 1 |  |  |  |  |  |
| **AurB IP enrichment** |  |  |  |  | 0.42 | 0.16 | 0.06 | -0.12 | -0.10 |
